# Effect of a Zinc Phosphate Shell on the Uptake and Translocation of Foliarly Applied ZnO Nanoparticles in Pepper Plants (*Capsicum annuum*)

**DOI:** 10.1021/acs.est.3c08723

**Published:** 2024-02-10

**Authors:** Sandra Rodrigues, Astrid Avellan, Garret D. Bland, Matheus C. R. Miranda, Camille Larue, Mickaël Wagner, Diana A. Moreno-Bayona, Hiram Castillo-Michel, Gregory V. Lowry, Sónia M. Rodrigues

**Affiliations:** † Centre for Environmental and Marine Studies (CESAM), Department of Environment and Planning, Universidade de Aveiro, 3810-193 Aveiro, Portugal; ‡ Centre for Environmental and Marine Studies (CESAM), Department of Chemistry, Universidade de Aveiro, 3810-193 Aveiro, Portugal; § Géosciences-Environnement-Toulouse (GET), CNRS, UMR 5563 CNRS, UT3, IRD, CNES, OMP, 31400 Toulouse, France; ∥ Department of Civil and Environmental Engineering, Carnegie Mellon University, Pittsburgh, Pennsylvania 15213, United States; ⊥ Centre de Recherche sur la Biodiversité et l’Environnement (CRBE), Université de Toulouse, CNRS, IRD, Toulouse INP, Université Toulouse 3 – Paul Sabatier (UT3), 31400 Toulouse, France; # The European Synchrotron, ESRF, 71 Avenue des Martyrs, CS40220, 38043 Grenoble, Cedex 9, France

**Keywords:** Capsicum annuum, micronutrient foliar delivery, phloem loading, spICP-TOFMS, nanoparticle persistence, micro X-ray fluorescence, micro X-ray absorption near-edge structure, Zn speciation, Zn cellular distribution

## Abstract

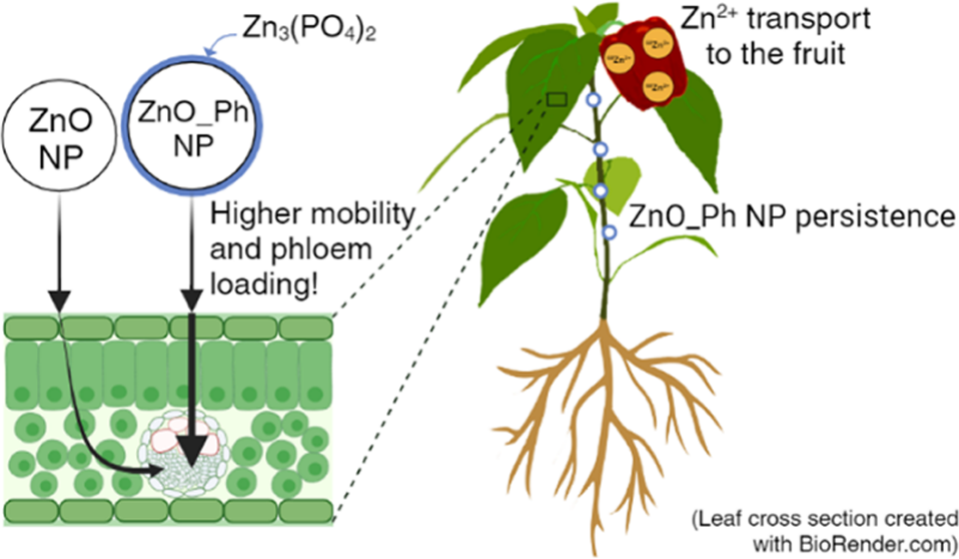

Here, isotopically labeled ^68^ZnO NPs (ZnO NPs) and ^68^ZnO NPs with a thin ^68^Zn_3_(PO_4_)_2_ shell (ZnO_Ph NPs) were foliarly applied (40 μg Zn) to pepper plants (*Capsicum annuum*) to determine the effect of surface chemistry of ZnO NPs on the Zn uptake and systemic translocation to plant organs over 6 weeks. Despite similar dissolution of both Zn-based NPs after 3 weeks, the Zn_3_(PO_4_)_2_ shell on ZnO_Ph NPs (48 ± 12 nm; −18.1 ± 0.6 mV) enabled a leaf uptake of 2.31 ± 0.34 μg of Zn, which is 2.7 times higher than the 0.86 ± 0.18 μg of Zn observed for ZnO NPs (26 ± 8 nm; 14.6 ± 0.4 mV). Further, ZnO_Ph NPs led to higher Zn mobility and phloem loading, while Zn from ZnO NPs was stored in the epidermal tissues, possibly through cell wall immobilization as a storage strategy. These differences led to higher translocation of Zn from the ZnO_Ph NPs within all plant compartments. ZnO_Ph NPs were also more persistent as NPs in the exposed leaf and in the plant stem over time. As a result, the treatment of ZnO_Ph NPs induced significantly higher Zn transport to the fruit than ZnO NPs. As determined by spICP-TOFMS, Zn in the fruit was not in the NP form. These results suggest that the Zn_3_(PO_4_)_2_ shell on ZnO NPs can help promote the transport of Zn to pepper fruits when foliarly applied. This work provides insight into the role of Zn_3_(PO_4_)_2_ on the surface of ZnO NPs in foliar uptake and *in planta* biodistribution for improving Zn delivery to edible plant parts and ultimately improving the Zn content in food for human consumption.

## Abstract 2

A zinc phosphate shell on ZnO nanoparticles improves Zn foliar mobility in pepper plants by increasing phloem loading of both ionic Zn and ZnO_Ph nanoparticles, ultimately delivering ionic Zn to pepper fruits.

## 1 Introduction

Zinc is an essential nutrient for both plants and humans. It has an important role in plant metabolism,^1,2^ including enzymatic reactions, photosynthesis, maintenance of cell membrane integrity, and pathogen resistance.^1,3^ The daily Zn intake required to meet the nutrient requirements for humans is between 8 and 11 mg Zn, and the Zn content in food is of extreme importance to reach these values.^4^ Zn is important for reproductive and immune system functions in humans.^5^ Globally, Zn deficiency causes ∼28 million deaths annually, and it is the fifth most important cause of illness and disease in developing countries.^6^ Therefore, it is important to find ways to improve the Zn content in food.

Zn deficiency in agricultural soils lowers the nutritional quality of crops, so zinc fertilizers are applied. Only 30–50% of fertilizers applied to soils are available for plant uptake, and the remainder accumulates in soil, leaches to groundwater, or runs off to surface waters.^2^ Alkaline (pH > 7) soils can limit Zn bioavailability to plant roots due to precipitation into solid Zn phases (Zn(OH)_2_, ZnCO_3_, ZnO, or Zn_2_SiO_4_).^6−8^ Thus, foliar application of Zn fertilizers can overcome these limitations of soil-applied Zn fertilizers because they are less affected by soil pH and they provide targeted delivery to crop plants, mitigating negative impacts on soil quality and the ecosystem.^6,9^

The commonly used Zn fertilizers for foliar application are generally soluble forms of Zn, such as ZnSO_4_, ZnEDTA, or ZnCl_2_.^8,10^ However, multiple applications are often needed because low concentrations are applied to prevent leaf burn or because there is inefficient uptake due to volatilization or from being washed-off of the leaves.^11^ Foliarly applied Zn-based nanoparticles (NPs) have been shown to supply Zn^2+^ to plants in a controlled and sustained manner through slow dissolution and uptake of released zinc ions.^12,13^ The uptake of NPs into the leaf is dependent on NP properties, e.g., size, solubility, surface charge, coating composition, and the plant cuticle/surface properties.^12,14−18^ Leaf surface properties, such as stomata and trichome density, cuticle composition (polysaccharides, fatty acids, etc.), and thickness, have been shown to influence NP adhesion and uptake.^19−23^ In several cases, smaller NPs adhere more to the leaf surface and are taken up into the leaf more readily than the larger ones.^20,24^ However, larger ZnO NPs that were encapsulated in a mesoporous SiO_2_ shell (∼70 nm, −12.9 mV) had 3-fold higher uptake into tomato plants compared to smaller, uncoated ZnO NPs (∼20 nm, −10.2 mV),^15^ indicating that coating composition may be as or more important than size in regulating leaf interaction and uptake. Surface modification of ZnO NPs with a plant nutrient like phosphorus could also improve uptake and slow the ZnO NP dissolution to provide a controlled release of Zn^2+^ inside the plant.^15^ As Zn_3_(PO_4_)_2_ has relatively low aqueous solubility, it has been hypothesized that a Zn_3_(PO_4_)_2_ shell on ZnO NP would prevent ZnO NP dissolution.^25^ It is not known if the foliar application of ZnO NPs coated with a Zn_3_(PO_4_)_2_ shell would promote ZnO NP uptake or affect dissolution and translocation within the plants.

The biotransformation and systemic translocation of ZnO NPs *in planta* are not yet understood. A review by Avellan et al.^14^ regarding foliar uptake and *in planta* translocation of inorganic NPs reported that out of 120 articles, only 16% discussed metal translocation to different plant compartments and that metal speciation *in planta* was usually not reported. A study by Ye et al.^26^ showed that foliarly applied Mn NPs crossed different plant tissues, accumulated in the leaf cuticle and upper epidermal cells, and were translocated to the spongy mesophyll. A better understanding of how NP properties influence foliar uptake pathways *in planta* transformation of metal-based NPs, phloem loading, and translocation of the element to the fruit can improve the design of nanobased formulations for foliar applications. Furthermore, studies that assess the persistence of ZnO NPs in plant organs over time are essential, especially in the edible parts of plants that present the potential for human exposure to NPs.

This study determined if (1) the amorphous zinc phosphate shell on ZnO NPs affected the foliar uptake of Zn compared to uncoated ZnO NPs, (2) if the foliarly applied NPs are taken up and translocated as NPs or zinc ions, and (3) if and where the NPs are persistent *in planta* over time. The effect of a Zn_3_(PO_4_)_2_ shell on Zn uptake, translocation, fate, and transformation over time was assessed by exposing pepper plants to either ZnO NPs or ZnO_Ph NPs. The results indicated that the Zn_3_(PO_4_)_2_ shell affected the uptake and distribution in the plants as well as Zn translocation to the fruits. The results obtained in this study suggest the possibility of tuning ZnO NP surfaces for the timely and targeted delivery of Zn inside the pepper plants by controlling Zn cell distribution, Zn *in planta* translocation, and ultimately delivering Zn to the pepper fruit.

## 2 Materials and Methods

### 2.1 ^68^ZnO-Based NP Synthesis and Characterization

The zinc oxide NPs enriched with ^68^Zn (ZnO NPs) were synthesized according to a method adapted from Wu et al.^27^ by primarily producing the precursor, Zn acetate (Zn_Act), enriched with ^68^Zn, as described by Dybowska et al.^28^ ZnO NPs with a Zn_3_(PO_4_)_2_ shell (ZnO_Ph NPs) were obtained by adapting the methods of Rathnayake et al.^25^ and Muthukumaran and Gopalakrishnan.^29^ For that, ZnO NPs were suspended in a pH 8 phosphate solution before being centrifuged and washed to recover for ZnO with Zn_3_(PO_4_)_2_ precipitated at the ZnO surface. Zn_3_(PO_4_)_2_ on the ZnO NPs is not a well-formed “shell”, but rather an amorphous and heterogeneously distributed precipitate of Zn_3_(PO_4_)_2_ at the surface of the ZnO NP core. Nanoparticle size was assessed by transmission electron microscopy (TEM) (Hitachi HT22700B) coupled to an energy dispersive spectrometer (EDS). The average NP size was evaluated by measuring the size of 150 particles using ImageJ software. The surface charge and hydrodynamic diameter of the nanoparticles were determined on 0.1 g/L suspensions using a Zetasizer Nano-ZS90 (Malvern Instruments, U.K.), and an average of 10 readings per sample were measured. Attenuated total reflection-Fourier transform infrared spectroscopy (ATR-FT-IR) was performed to analyze the NP surface on an Avatar 360 Thermo Nicolet spectrometer and expressed as an average of 64 readings. X-ray diffraction (XRD) analysis standards were obtained with Cu Kα radiation using an Empyrean diffractometer (PANalytical, The Netherlands). The XRD data were analyzed using Match 3 (PANalytical BV Almelo, The Netherlands) for the identification of the crystalline phases.

The total zinc content was determined by inductively coupled plasma mass spectrometry (ICP-MS, Thermo-X Series), and details regarding quality control procedures can be found in the SI. The samples were digested in a microwave (Speedwave 4, Berghef) (Table S1) as described by Martins et al.,^2^ and measurements were performed in triplicate.

All details regarding both ZnO NP and ZnO_Ph NP synthesis methods and characterization technical details can be found in the SI.

### 2.2 Dissolved ^68^Zn Release from ZnO NPs and ZnO_Ph NPs in Milli-Q Water and Simulated Phloem Sap

^68^Zn^2+^ release from ZnO NPs and ZnO_Ph NPs was assessed in Milli-Q water (MQ water) and in simulated phloem sap. The simulated phloem sap solution was prepared according to previous studies and contained the following dissolved constituents: sucrose (90 mM), serine (11.4 mM), aspartate (9.1 mM), KCl (15 mM), CaCl_2_ (1.5 mM), MgSO_4_ (1.5 mM), NaCl (5 mM), and HEPES (10 mM).^30−32^ The pH of the simulated phloem sap was 7.0 ± 0.1,^32^ and the composition used in this study can be found in the SI (Table S2). In the present study, 3 mg L^–1^ of Zn suspensions of each nanomaterial was prepared in either MQ water or simulated phloem sap in 50 mL tubes. The tubes were laid horizontally on a reciprocating shaker (150 rpm) in the dark for the entire duration of the test. At several time points, over 3 weeks for MQ water (0, 1, 2, 24, 168, and 504 h) and 1 week for simulated phloem sap (0, 1, 2, 24, and 168 h), 2 mL aliquots were taken from the tubes and centrifuged for 30 min at 16,392*g* (Eppendorf 5415R, rotor: F-45–24–11). The supernatant (the top 0.5 mL) was then diluted with MQ water, acidified with 2% v/v HNO_3_, and analyzed by ICP-MS. The zeta potential was measured on the initial suspensions using a Zetasizer Nano-ZS90 (Malvern Instruments, U.K.), and the average of 3 readings per sample was measured at each time point of the dissolution experiments.

### 2.3 Pepper Seed Germination and Plant Growth

Pepper seeds (*Capsicum annuum* L.) were obtained from Johnny’s Selected Seeds (https://www.johnnyseeds.com/). The seeds were soaked in deionized water (DIW) overnight (8 h), gently shaken for 2 min in a 5% v/v bleach solution for surface sterilization, and then rinsed with DIW to remove all traces of bleach (10 times). Seeds were then placed on DIW-moistened towel paper in a Petri dish and kept in a growth chamber with a light/dark cycle of 16 h/8 h (25 °C/21 °C and 60% humidity) for 7 days for germination. Pepper seedlings were transferred into a 60 mL syringe filled with silica sand (ACROS Organics). Pepper plants were used as model plants for growth chamber tests, which allowed the development of fruits in a short time frame. The silica sand was previously washed with DIW, followed by acid washing (5% v/v HNO_3_) overnight, rinsed with DIW, dried at 90 °C for 24 h (for water evaporation), burned at 250 °C overnight to remove salicylic acid, and finally rinsed thoroughly with DIW. A sand substrate was used in this study because of the possibility of acid washing that removed all metals present in the sand matrix, allowing for controlled nutrient delivery to plants with a Zn-free 1/4 Hoagland solution. The sand substrate also provided the plant roots with a solid structure that allowed for a similar morphological development as if they had been grown in soil.^33^ A rope made of 100% cotton was introduced to connect the sand in the syringe to a nutritive solution below the syringe to maintain the sand humidity over time through capillary exchange. A 1/4 strength Hoagland solution (Table S3) was used as a nutritive solution, which was prepared without Zn to ensure that the plants would not go through deficiencies other than Zn.^34^ Plants were grown in the growing chamber under the same environmental conditions as for the seed germination detailed above, for the total duration of the experiment (12 weeks).

### 2.4 Pepper Plants Exposure to Zn and Harvesting

Plants were exposed in the sixth week of growth, and the materials were applied on 2 leaves per plant (6th and 7th leaves). Each plant was exposed to a total of 40 μg of Zn. For this purpose, 270 μL was foliarly applied by drop deposition on the adaxial side of the leaves with a pipet (13 × 10 μL + 1 × 5 μL per leaf) of 150 mg Zn/L as ZnO NPs, ZnO_Ph NP suspensions, Zn salt, or DIW (for the negative control). All treatments (including the DIW control) were dispersed with 0.1% v/v Silwet L-77 (PhytoTech laboratories, Inc.) to enhance foliar uptake by disrupting the cuticle wax layer.^21,35^ It should be noted that the Silwet L-77 surfactant improved leaf surface wettability, allowing for an even distribution of foliarly applied treatments. The same dose used in this study (40 μg of Zn per plant) has been used previously in tomato plants (*Solanum lycopersicum*).^15^ This dose was also chosen here to provide sufficient Zn while avoiding toxicity to the pepper plants. The sufficient Zn levels in leaves have been reported to vary between 21 and 120 μg/g dry mass.^36^ The Zn control used in this study (Zn salt) was prepared by acidifying a ZnO NP suspension (150 μg Zn/L) with 1 M HCl to pH 2 for 24 h and then raising the pH back to pH 7.2 ± 0.2 by using a 1 M NaOH solution. Four plants per treatment were harvested at different time points: 1 week, 4 weeks (flowering stage), and 6 weeks (fruiting stage) after exposure, and separated by compartment: both exposed leaves, all the remaining leaves, stems, and roots. The roots were briefly submerged in MQ water to remove any attached sand. Four plants were used in each condition. For all plants (even the control plants), both exposed leaves fell off approximately 2 weeks after exposure, and no more Zn uptake could occur. The duration of the experiment was 12 weeks since exposure was performed at the sixth week of growth, and the oldest plants (fruiting stage) were harvested 6 weeks after exposure.

### 2.5 Assessment of Zn Adhesion to Pepper-Exposed Leaves by Wash-Off Tests

For plants that were harvested 1 week after exposure, the exposed leaves were cut and rinsed to assess both the loosely and strongly adhered fractions of the applied materials (there were no exposed leaves sampled at the other time points due to the leaves falling off). To assess the loosely attached fraction of the materials applied, a washing-off solution made of soluble salts was prepared as described by Kah et al.^37^ (the composition can be found in Table S4). Each of the exposed leaf was cut from the plant and washed with 50 mL of the washing solution in a 50 mL centrifuge tube for 9 s. Afterward, the leaves were dipped for 3 s in another solution containing 2% HNO_3_ and 3% ethanol to remove Zn strongly attached to the surface of the exposed leaves.^37^ Since no nutrient leakage occurred in the DIW-exposed leaves wash-off, the integrity of the leaf cells prior to rinsing was maintained. Two exposed leaves per plant were washed together. Four plants per treatment were subjected to this process. All solutions were acidified/diluted with 1% HNO_3_ before analysis by ICP-MS (Agilent 7700).

### 2.6 Analysis of Total Zn Concentration Inside Pepper Plant Tissues by Microwave Digestion and ICP-MS Analysis

The exposed leaves, remaining leaves, stems, and roots were oven-dried at 60 °C for 48 h and then microwave-acid digested. Both the seeds of*C. annuumL*. and sand were also digested to assess the Zn background level (Tables S5 and S6). The detailed digestion protocol used here can be found in the SI. All tissues of four plants per condition were digested, and all digestates were diluted with 1% HNO_3_ before analysis by ICP-MS (Agilent 7700).

The total Zn recovery was assessed on plants harvested 1 week after exposure, for all applied materials, by summing the ^68^Zn analyzed in the washing-off solution of the leaves, plus the ^68^Zn mass in the digestate of exposed leaves, remaining leaves, stems, and roots. The uptake of ^68^Zn was calculated as the ^68^Zn that was not washed off from the exposed leaves, i.e., the sum of ^68^Zn that was measured from the digestates of the exposed leaves (after the washing-off), the remaining leaves, stems, roots, and fruits, after subtracting the ^68^Zn in the DIW control.

### 2.7 Recovery of Zn NPs in Pepper Plant Tissues by Methanol (MeOH) Digestion

To assess the possibility of NP foliar uptake, translocation, and possible persistence in other plant organs, a methanol-based digestion protocol from Laughton et al.^38^ was used. This protocol has been shown to be effective in the recovery of ZnO NPs in plant tissues while maintaining some stability for possible dissolution. The dilution for single-particle inductively coupled plasma time-of-flight mass spectrometry (spICP-TOFMS) was performed immediately prior to the analysis. Bearing this in mind, the protocol was performed for all plant organs, as described by Laughton et al.,^38^ and can be found in the SI. Three plants per condition were subjected to this process and analyzed by spICP-TOFMS. For spICP-TOFMS analysis, MeOH extraction samples (pH 9) were diluted with DIW and bath-sonicated for 5 min immediately prior to measurement to prevent any possible risk of NP dissolution. This analysis was based on the number of particle events rather than particle concentration. The observed particle events are ZnO NPs because the NPs used are 98% enriched with ^68^Zn. We used spICP-TOFMS to qualitatively confirm the presence of particles in some tissues. While we have used this method previously to quantify ZnO NPs in different plant tissues,^38^ here, it has been used only qualitatively. The approximate size cutoff for ZnO NPs is about 40–50 nm, as shown by Laughton et al.^38^; therefore, particles smaller than this would not be detected. This has been added to the discussion on the spICP-TOFMS results. Further details regarding the TOF detector and instrument parameters can be found in the SI.

### 2.8 Zn Distribution and Speciation on Pepper Fresh Tissues Using Micro X-ray Fluorescence (μ-XRF) and Micro X-ray Absorption Near-Edge Structure (μ-XANES)

Both μ-XRF maps and μ-XANES (recorded above the Zn K-edge −9.65 keV) measurements were performed using the scanning X-ray microscope at the ID21 beamline of the European Synchrotron Radiation Facilities (ESRF, Grenoble-Fr).^39^ Nonwashed exposed leaves (2 h and 1 week after exposure) and stems (1 week after exposure) were sampled, embedded in optimal cutting temperature (OCT), and flash-frozen in liquid nitrogen. Cross sections of 20 μm thick were done under cryogenic conditions (−170 °C) using a cryomicrotome at the beamline, and cross sections were transferred, still frozen, on the cryostage of the beamline. The 2 h and 1 week time points on the exposed leaves were chosen to assess the temporal evolution of Zn cellular internalization and speciation within the plant-exposed leaf, and the 1 week postexposure in the stems was chosen to assess the translocation of the Zn NPs and zinc speciation. All μ-XRF maps (at 9.8 kev) and μ-XANES scans were obtained under cryo-conditions and in vacuum. The compounds for the XANES references were synthesized according to the literature (Table S7). The details of the reference preparation are detailed in the SI. The averaged XANES spectra obtained for each reference are shown in Figure S1 in the SI.

Data from μXRF were processed using PyMCA software (version 5.8.1).^40^ All raw data were dead time-corrected and normalized by the incoming intensity and fitted in PyMCA to obtain elemental distribution maps that were then overlaid as RGB images. Orange software (version 3.35.0) with the spectroscopy add-on was used to perform principal component analysis (PCA) on second-derivative XANES spectra.^41,42^ These PCAs were used to obtain the average XANES from several representative points of interest (POIs). Larch software (version 0.9.68)^43^ was used for spectral normalization and linear combination fitting (LCF).

### 2.9 Statistical Analysis

The data were analyzed using IBM SPSS Statistics (Version 29.0). Significant statistical differences between total uptake and translocation of ^68^Zn were assessed using one-way ANOVA analysis (*p* < 0.05 threshold) between the different Zn treatments and the no Zn control treatment.

## 3 Results and Discussion

### 3.1 Nanoparticle Surface Functionalization and Abiotic Reactivity

The ZnO NP and ^68^ZnO_Ph NP both had diffraction peaks at 2θ = 31.8, 34.4, 36.3, 47.6, and 56.7°, which are characteristic of the crystalline hexagonal ZnO NP (Figure S2).^25,44^ However, the addition of an amorphous and heterogeneously distributed Zn_3_(PO_4_)_2_ shell reversed the particle charge in MQ water (pH = 6.8) from +14.6 mV for ZnO NP to −18.1 mV for ZnO_Ph NP (Table 1). This phosphate layer will be referred to as the shell in this manuscript. This is consistent with the observations of Baddar and Unrine,^45^ who reported that the Zn_3_(PO_4_)_2_ shell on ZnO NPs lowered the pH_pzc_ from 9.8 for ZnO to <6.2. The TEM particle size distribution (Figures S3 and S4) showed that the ZnO_Ph NP (48 ± 12 nm) was also larger than ZnO NP (26 ± 8 nm). Furthermore, no significant differences in shape were observed between the ZnO NPs and ZnO_Ph NPs, and the only difference observed was the presence of a heterogeneous amorphous Zn_3_(PO_4_)_2_ layer on the surface of the ZnO_Ph NPs. EDS and FT-IR analyses confirmed the presence of P on the surface of the ZnO NP (Figures S4 and S6).

**Table 1:** ZnO NP and ZnO_Ph NP Properties

	medium	TEM average nominal size (nm)^a^^,^^b^	ζ potential (mV)^c^	hydrodynamic diameter (nm)^b^^,^^c^	Zn (%w/w)^c^	pH of the medium
ZnO NP	MQ water	26 ± 8	14.6 ± 0.4	357 ± 126 (0.34 ± 0.02)	89.9 ± 6.7	6.8 ± 0.2
	simulated phloem	N/A	–7.5 ± 1.3	74 ± 6 (0.31 ± 0.03)	N/A	7.2 ± 0.1
ZnO_Ph NP (2.0 ± 0.1% P)^c^	MQ water	48 ± 12	–18.1 ± 0.6	317 ± 87 (0.59 ± 0.07)	83.6 ± 1.1	6.8 ± 0.2
	simulated phloem	N/A	–6.0 ± 1.6	118 ± 6 (0.44 ± 0.01)	N/A	6.9 ± 0.1

a Based on the TEM images of at least 150 particles.

b Intensity-weighted Z-average.

c The results are presented as mean ± standard deviation (*N* = 10 for ζ potential; *N* = 3 for Zn and P%). %w/w – % as weight/weight. PDI values for the hydrodynamic diameter are presented in parentheses. N/A – not applicable.

The charge and dissolution rate of the particles in the simulated phloem were measured and compared with those in MQ water (Figures S7 and S8). Both Zn-based NPs had ∼20% dissolution after 1 week (168 h) in MQ water and simulated phloem sap and ∼30% dissolution after 3 weeks (504 h) in MQ water. It is worth mentioning that for both Zn-based NPs, dissolution occurred at time 0 h in either MQ water or phloem sap (7–10 and 20%, respectively). Rathnayake et al.^25^ suggested that the Zn_3_(PO_4_)_2_ shell could protect the ZnO core, slowing dissolution. However, in our study, no significant difference was observed in the dissolved Zn measured for both Zn-based NPs in MQ water or in simulated phloem sap over 3 weeks (Figures S7 and S8). It is possible that the Zn_3_(PO_4_)_2_ shell was too thin or not uniform enough to prevent the ZnO core from dissolving. Moreover, the differences in nominal sizes of the NPs did not affect the rate of dissolution.

The ZnO_Ph NPs were negatively charged in both MQ water and in the simulated phloem sap. However, the charge of uncoated ZnO NPs changed from positive 14.8 mV in MQ water to −7.5 mV in simulated phloem (Table 1). This is likely because of electrostatic interactions between the positively charged ZnO NP and chloride, sulfate, and negatively charged amino acid (aspartate) present in the simulated phloem sap at pH 7.

### 3.2 ^68^Zn Foliar Uptake and *In Planta* Translocation

No statistically significant differences were observed in plant dry biomass between Zn-based NPs and the Zn salt treatments (Figure S9), except 4 weeks after exposure, in which the Zn salt treatment had lower total dry biomass (*p* < 0.05) when compared to both Zn-based NPs. The Zn salt treatment did have a significantly lower dry biomass than the nonexposed control at all time points.

**Figure 1:**
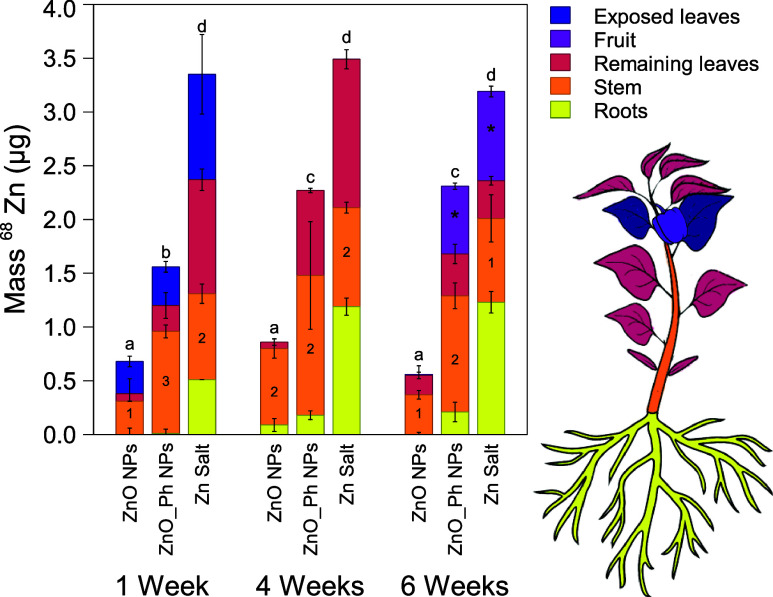
^68^Zn mass in each plant organ. The ^68^Zn in the nonexposed controls was subtracted from all treatments. Error bars represent the weighted standard deviation of the samples from four replicate plants. Statistically significant differences (*p* < 0.05) of the means of total ^68^Zn masses between treatments are indicated by different letters (on top of each bar chart). Numbers and asterisks inside each bar represent statistical significance for the stem and the fruit, respectively.

At all time points after foliar application, the total ^68^Zn uptake was lower for both Zn-based NPs than for the ^68^Zn salt. However, the total ^68^Zn uptake by plants exposed to ZnO_Ph NPs was 2.3–4.2 times higher than that of plants exposed to ZnO NPs (Figure 1). Tomato plants foliarly exposed to ZnSO_4_^46^ and ZnCl_2_^15^ have been reported to take up more Zn than those exposed to ZnO NPs. A study in wheat^12^ also found that foliarly applied Zn salt and chelated Zn forms provided more Zn^2+^ uptake than ZnO NPs due to limited particle dissolution and, therefore, lower available amounts of Zn^2+^ for absorption. It is, however, important to note that Zn could also be taken up as NPs. Therefore, differences in the properties like surface chemistry of ZnO NPs and ZnO_Ph NPs may also have caused differences in the uptake of Zn as NPs.

The dissolution of both Zn-based NPs was similar in MQ water (representing the leaf dosing condition); therefore, differences observed in the total ^68^Zn uptake for the Zn-based NPs cannot be attributed to differences in the dissolution rate. Moreover, the size was also not decisive. Smaller NPs have been reported to adhere to the leaf surface better, dissolve faster, and take up more than larger ones^20^; however, here, the highest uptake was observed for the larger ZnO_Ph NPs. A similar observation was made in the uptake by tomato plants of ZnO NPs coated with a mesoporous SiO_2_ shell.^15^ Thus, either the charge or the Zn_3_(PO_4_)_2_ shell promoted the higher uptake of Zn from ZnO_Ph NPs than ZnO NPs.

The ZnO and ZnO_Ph NPs have opposite surface charges in MQ water. The differences observed for ^68^Zn uptake for the tested NPs could be due to the lower affinity of the negatively charged ZnO_Ph NPs to the pepper leaf cells (also negatively charged^14,35^), promoting uptake into stomata or through the cuticle. The different functional groups and associated surface charge changes might also modulate the capacity for biocorona formation and further translocation mechanisms. It is also possible that the differences observed were due to the phosphate groups (Zn_3_(PO_4_)_2_) on the ZnO NPs, which could lead to differences in cellular recognition and, therefore, differences in translocation once inside the leaf. Higher translocation away from the site of entry may promote additional uptake.

The ^68^Zn distribution inside the pepper plants (Figures 1 and S12) for both Zn-based NPs was different from that of the Zn salt. At all time points, pepper plants exposed to Zn salt had significantly more ^68^Zn mass translocated to the roots than those exposed to ZnO NPs. ZnO NPs had a maximum of ∼10% of the total ^68^Zn mass translocated to the roots. For both Zn-based NPs, ^68^Zn mass was predominantly found in the stem. Between the fourth and sixth weeks after exposure to Zn salt, ^68^Zn that had been translocated to the nonexposed leaves of plants decreased, apparently translocating to the fruits. In ZnO NP-exposed plants, ^68^Zn translocated from the stem to the upper leaves by the sixth week, but there was only a minor translocation of ^68^Zn to the fruit (0.03% of the total ^68^Zn uptake). However, in plants exposed to ZnO_Ph NPs, ^68^Zn that had been primarily translocated to the stem (when compared to the Zn salt) was translocated to other leaves and to the fruit (27% of the total ^68^Zn uptake) after 6 weeks. Zn salt and ZnO_Ph NPs led to an enrichment in ^68^Zn in the fruits (0.63 ± 0.03 μg for ZnO_Ph NPs and 0.83 ± 0.05 μg for the ^68^Zn salt treatment). This was significantly higher than for bare ZnO NPs, which only led to 0.01 ± 0.08 μg of ^68^Zn in the pepper fruit. Both Zn salt and ZnO_Ph NPs foliarly applied to pepper plants led to a concentration of 2 mg Zn/kg in pepper fruits, which is the amount of Zn that has been reported by the USDA in the nutritional content in red bell peppers (2 mg/kg).^47^ Even though more Zn was translocated to the fruits of pepper plants exposed to Zn salt and ZnO_Ph NPs, the fruit biomass was not statistically significant between the three treatments (Figure S9). The different ^68^Zn allocations inside the plants and the changes observed in ^68^Zn allocation over time suggest that there was a different Zn transport pathway involved after uptake.

Both ZnO and ZnO_Ph NPs were negatively charged in the simulated phloem (Table 1), suggesting that phosphate, rather than charge, affected translocation. There is a systemic regulation of inorganic phosphate (Pi) in plants, and crosstalk signaling between Pi and Zn has been reported.^48−50^ In fact, when plants face Zn deficiency, the transcription of Pi-related genes is activated, increasing Pi uptake.^48^ Our plants were grown in a Zn-deficient system, i.e., no Zn was added to the nutrient solution, and the sand was acid-washed to remove Zn. Thus, when exposed to ZnO_Ph NPs, the plant could have upregulated phosphate translocation and, therefore, Zn as well.

### 3.3 ^68^ZnO NP Persistence over Time

**Figure 2:**
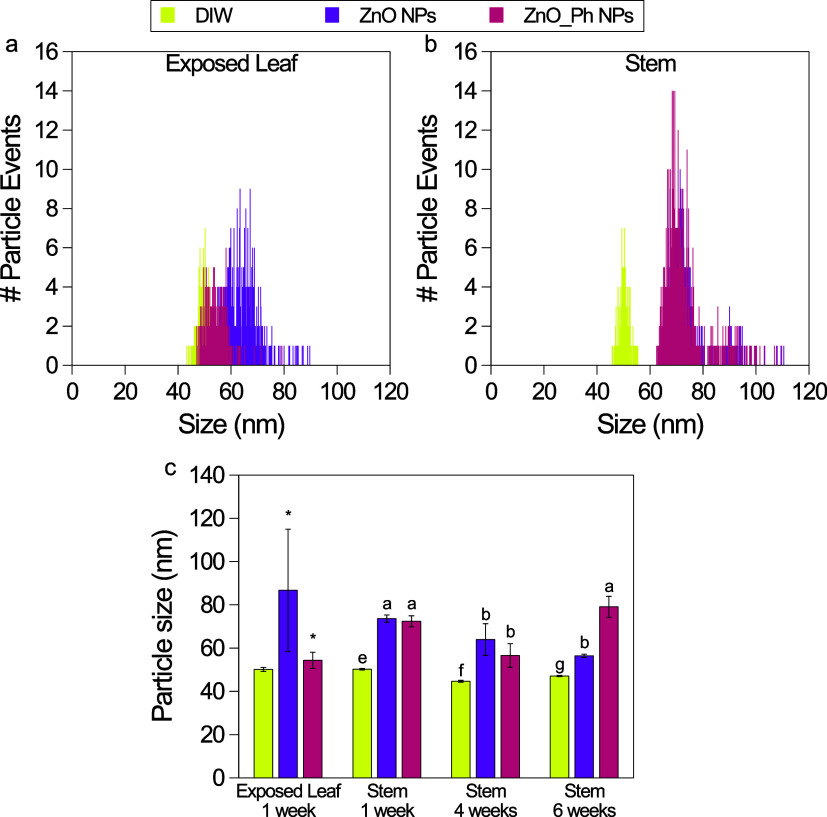
Particle size distribution of NPs containing ^68^Zn measured 1 week after exposure by spICP-TOFMS in exposed leaves (a) and stems (b). Weighted average of the particle sizes detected by spICP-TOFMS in the exposed leaves (after washing) and in stems at 1, 4, and 6 weeks after foliar exposure (c) of pepper plants exposed to ZnO NPs (purple), ZnO_Ph NPs (pink), and in control plants (DIW; yellow). The DIW control contained naturally occurring ^68^Zn particles. Error bars represent the weighted standard deviation of the samples from three replicate plants. Statistically significant differences (*p* < 0.05) between treatments in exposed leaves are indicated by asterisks and in stem by different letters.

Our spICP-TOFMS results showed that both Zn-based NPs were present 1 week after exposure to the washed exposed leaves. These were also present in stems at 1, 4, and 6 weeks after exposure (Figure 2). For both the washed exposed leaves and stems, NP treatments showed significantly larger ^68^Zn particles than DIW-treated plants (*p* < 0.05). Also, no other elements were associated with these ^68^Zn particle events, indicating that the observed particle signals came from the applied nanomaterials. ^68^Zn is a naturally occurring Zn isotope (18.8%), which is why some ^68^Zn particles were naturally present in the DIW control. However, the ^68^Zn particle counts present in the remaining leaves, roots, and fruits of the Zn-based NP-treated plants were not significantly different from those detected in the DIW control (Figure S13). These results indicate that the applied materials were not likely to translocate as NPs to these organs; however, due to the size cutoff (40–50 nm), this cannot completely rule out the possible translocation of smaller NPs to the other organs.

The particle size of ZnO NPs in the stem appears to decrease with time over a period of 6 weeks (Figure 2c). The dissolution of ZnO NPs in tomato plants foliarly exposed to nZnO@SiO_2_ NPs has been reported by Gao et al.^15^ The SiO_2_ NPs that were detected by spICP-MS were reported to decrease in all plant organs where NPs were detected due to dissolution related to plant metabolism once inside the plant. In contrast, the sizes of the ZnO_Ph NPs in the stem over time are more consistent. This contrasts with dissolution studies that showed that both particles dissolved at the same rate. Even though the overall dissolution of ZnO_Ph NPs inside the stem over time is apparently lower than that of ZnO NPs, there was more ^68^Zn being translocated to the remaining leaves in the fourth week, translocating from the remaining leaves to the fruit in the sixth week. Understanding the form and speciation of Zn inside the pepper plants upon foliar uptake is therefore needed to provide more insight into the cellular mechanisms involved in Zn allocation, transport, and/or sinking in specific plant cells.

### 3.4 Zn *In Planta* Mobility

**Figure 3:**
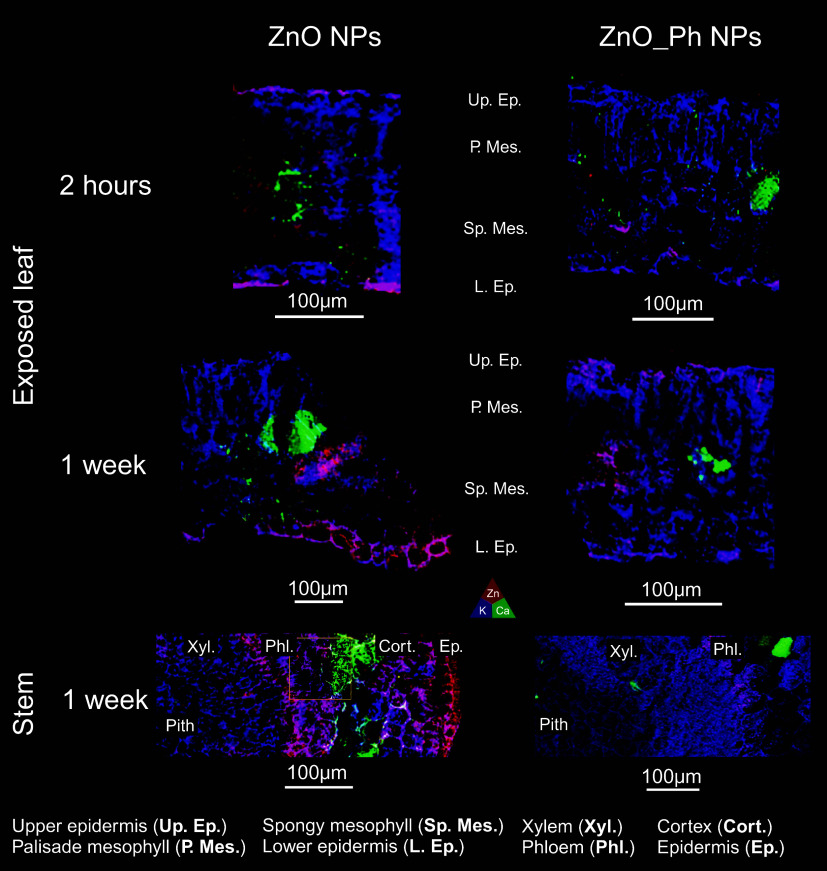
Elemental μ-XRF maps of the seventh leaf of the pepper plant exposed to ZnO_Ph NPs and ZnO NPs: *2* h after exposure (top row); 1 week after exposure (middle row), and on the pepper plant stem near the seventh leaf node 1 week after exposure (bottom row). The fluorescence signals of Zn, K, and Ca are represented in red, blue, and green, respectively.

Both the nanoform of Zn-based NPs and the time affected the location of Zn in the leaves and stems (Figure 3). It is worth mentioning that the Zn signal was below the detection limit in the unexposed plant leaves and stems (Figure S14). μ-XRF was not used here to quantitatively compare the NP treatments. Pepper plant leaves exposed to ZnO NPs and ZnO_Ph NPs had Zn in the upper epidermis, palisade, spongy mesophyll, and lower epidermis 2 h and 1 week after exposure. The signal was less intense for the ZnO_Ph NPs than for the ZnO NPs. ZnO NP treatment led to a high allocation of Zn in the lower epidermis cell walls and their cytosol 1 week after exposure. Other studies using ZnO NPs,^51^ Mn NPs,^26^ and Ag NPs^52^ foliarly applied to wheat seedlings, pepper plants, and lettuce similarly showed NP accumulation in the upper epidermis, spongy mesophyll, and palisade mesophyll of the exposed leaves. The sections of the stem immediately below the exposed leaf petiole were used to study Zn that reached the phloem and was mobilized to other parts of the plant. μ-XRF revealed that Zn was accumulated in the stem epidermis and cortex inward of the vasculature of ZnO NPs. In contrast, Zn accumulation was only observed in the vasculature of ZnO_Ph NPs at the same stem location (Figure 3).

Zinc accumulation on the leaf lower epidermis cell wall, stem epidermis cell wall, and cortex in ZnO NP-exposed plants suggests that this could be a storage strategy or potentially an excretory and/or detoxification system for Zn when exposed to ZnO NPs.^53,54^ Ye et al.^26^ suggested that autophagy of Mn NPs in the exposed pepper leaves, as well as accumulation in intracellular spaces, occurred. It has also been demonstrated that plant cell walls and the root cortex can accumulate trace metals, such as Zn.^55,56^ In contrast, for ZnO_Ph NPs, we did not find the same pattern of Zn storage in the epidermis; rather, it appears that Zn was mainly phloem-loaded upon uptake. These results indicate that there could be a different mechanism of allocation and storage of Zn in pepper plant leaves upon uptake and/or that they presented contrasting *in planta* transformation depending on the surface chemistry of NP.

XANES spectra were thus regarded at different points of interest (POIs) on each sample to further study Zn speciation in the samples. The number of POIs where XANES spectra were collected is shown in Table S8. The linear combination fitting (LCF) results obtained from the fitting of μ-XANES spectra done in different POIs on each cell tissue from the exposed leaves 2 h and 1 week after exposure and stem 1 week after exposure are shown in Table S9. LCF analysis of Zn K-edge XANES spectra showed that 2 h after exposure, some of the applied Zn remained nanoparticulate in the exposed leaves for both Zn-based NPs. For ZnO NPs, ZnO NP persistence was observed in both the upper epidermis and palisade mesophyll, while the presence of ZnO_Ph NP was mainly observed in the palisade and spongy mesophyll, with few NPs detected in the upper epidermis. For longer exposure times (1 week), contrary to the spICP-TOFMS results, μ-XANES did not show any evidence of ZnO NPs in the exposed leaves of ZnO NP-treated plants or in the stem vasculature for either of the tested NPs. It should be noted that though we only performed XANES spectra on selected points of interest (POIs), these results do not exclude the presence of NPs that could have just been missed. The Zn speciation described next provides more insight into how speciation is affected by the different routes of cellular translocation involved.

**Figure 4:**
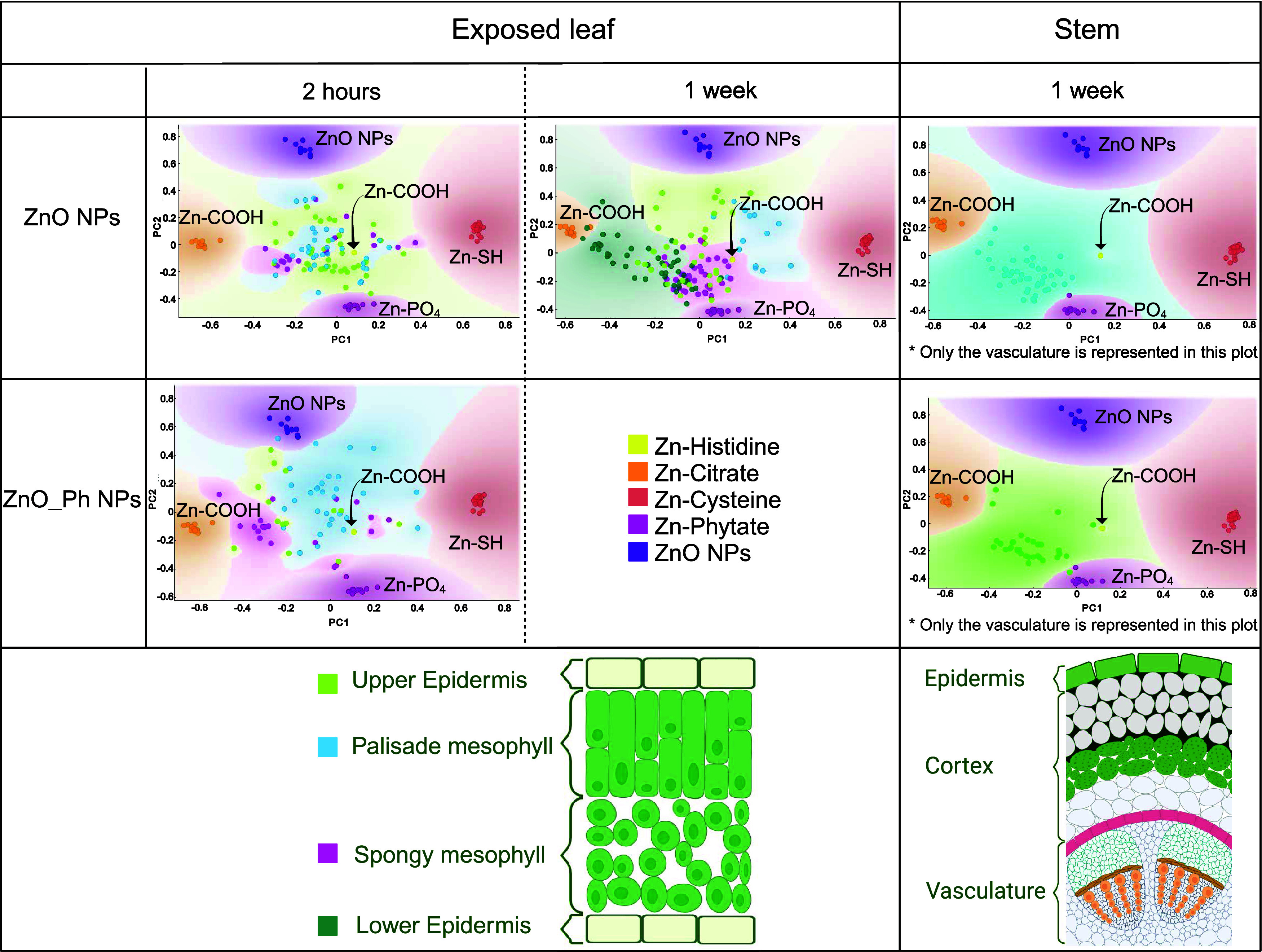
PCA plots of the XANES spectra done on selected POIs in selected leaf compartments for leaves exposed to ZnO NPs (2 h and 1 week after exposure) or ZnO_Ph NPs (2 h after exposure), and stems of plants exposed to ZnO NPs and ZnO_Ph NPs (1 week after exposure). Linear combination fitting of the groups of these POIs can be found in the Supporting Information (Table S9) (leaf and stem scheme created with BioRender.com).

Zinc speciation was analyzed on points of interest (POIs) on selected Zn hotspots and are presented as PCA plots relative to different Zn references (Figure 4 and Table S9). In these PCAs, the closer a sample POI is to the reference XANES spectra, the more alike the XANES spectra are. ZnO-based NPs were transformed differently in various cellular compartments and over time. After 2 h of exposure, POI in leaves exposed to ZnO NPs or ZnO_Ph NPs indicates that Zn speciation did not seem to be driven by the compartment the POI was obtained from (upper epidermis cells, palisade, or spongy mesophyll), as the POI from various compartments did not cluster together. For both treatments, Zn was primarily associated with the carboxyl (COOH) and phosphate (PO_4_) groups. Proteins containing carboxyl groups (such as histidine-rich proteins) are known to be binding sites for Zn in the cell wall.^57^ This suggests that Zn that was bound to carboxyl groups was present in the cell walls. Regarding ZnO_Ph NPs, 2 h after exposure, more POIs showed that Zn was associated with thiol-like groups in the upper epidermis and palisade mesophyll. This suggests that Zn could be associated with metalloproteins. Such types of proteins have been reported to participate in Zn phloem loading^57^ and to chelate Zn, participating in Zn sequestration in the phloem.^58^ Phytate and organic acids containing carboxyl groups, such as citrate, have been reported to be bound to Zn in plant cell vacuoles after metal tolerance proteins transport Zn inside vacuoles.^58,59^ Altogether, these results suggest that after 2 h of exposure, Zn from ZnO_Ph NPs was slightly more mobile than the Zn from ZnO NPs. The presence or absence of phosphate on the NPs seems to cause the differences observed between NPs in both translocations inside the plant and cellular internalization of Zn upon uptake. Such differences are expressed by differences in Zn speciation depending on Zn localization in cells and time after exposure.

One week after exposure to ZnO NPs, Zn speciation seemed much more variable depending on the compartment from which μ-XANES spectra were obtained as POIs clustered together (Figure 4). First, no ZnO NP appeared to remain in the exposed leaves. Zn from the upper epidermis is still mainly associated with carboxyl groups. Some of the Zn taken up in the palisade mesophyll was found to be associated with thiol groups, while Zn from the spongy mesophyll was partly associated with phosphates. In the lower epidermis, where a lot of the Zn was found (Figure 3), Zn was mainly associated with carboxyl groups, indicating Zn binding to the cell wall.^57^ Zinc speciation in the vasculature area in the stems of plants exposed to both Zn-based NPs showed speciation similar to that in the lower epidermis of the cells.

When looking at the speciation of Zn that reached the stem (in the stem phloem), Zn appeared to have similar speciation in both treatments. Zn was bound to phosphate and carboxyl groups, indicating both a possible allocation to the vacuoles^58^ and Zn binding to the cell wall,^57^ respectively.

Overall, the above-described results indicate that Zn from ZnO_Ph NPs presented a higher mobility than that from ZnO NP treatment in the exposed leaves, which is correlated with Zn associated with carboxyl and thiol groups. A large amount of Zn from ZnO NPs was stored in the epidermis layers, in both the leaves and the stem, while we did not observe this in the ZnO_Ph NP-treated samples. Finally, Zn reaching the stem vasculature had similar speciation, regardless of the initial treatment.

### 3.5 Benefits of the Zn-Phosphate Shell for ZnO NP Phloem Loading

The distinct surface coatings of ZnO NPs and ZnO_Ph NPs triggered different plant strategies for Zn translocation and storage when they were foliarly applied to pepper leaves. Zinc was translocated to the stem both as ionic Zn and as NPs in both treatments, but there was a different plant strategy for storage/sinking for ZnO NPs. Zn accumulation in both the leaf and stem epidermis observed only in plants exposed to noncoated ZnO NPs suggests that the Zn_3_(PO_4_)_2_ shell on ZnO NPs promoted faster phloem loading of Zn, which consequently led to higher Zn translocation to the nonexposed leaves (at early stage) and further to the pepper fruit. Zinc transport to the fruit was not observed for noncoated ZnO NPs.

The results of this study also suggest that the stem is a Zn-accumulating organ for plants dosed with both Zn-based NPs, which was not observed for the Zn salt. The NPs containing Zn were detected in the stem over 6 weeks after application in the ZnO_Ph-treated plants. However, some dissolution of ZnO_Ph NPs seemed to occur in the stem over time. Zn was mostly allocated to the vasculature rather than to other cell tissues, which corroborated the phloem loading. This could also explain the transport of Zn to the remaining leaves and the subsequent transport of ionic Zn to the pepper fruit. No NPs were detected in the pepper fruits.

These findings support the possibility of using a Zn_3_(PO_4_)_2_ shell on ZnO NPs for more efficient foliar uptake of ZnO NPs by stimulating phloem loading through associations with amino acids promoting NP dissolution inside the plant and targeting Zn delivery to pepper fruits.

### 3.6 Environmental Implications

In our study, the Zn-phosphate shell enhanced ZnO NP uptake and NP storage inside the stem and promoted faster phloem loading when compared to uncoated ZnO NPs. The storage of ZnO NPs in the stems of ZnO_Ph NPs-exposed plants, coupled with slow dissolution over the plant growth cycle, facilitated Zn transport from the stem to other leaves and ultimately from other leaves to the fruit. However, in our study, the Zn-based NPs did not enhance fruit fortification when compared to the Zn salt, and the total dry biomass of the plants treated with the Zn salt was significantly lower compared to that of the nonexposed control. Although ZnO_Ph NPs translocated Zn to the fruit (26% of the Zn uptake), we did not find evidence of the presence of NP in the pepper fruits, which would avoid exposure from human consumption.

The Zn-phosphate shell induced differences in Zn translocation strategies (symplastic vs apoplastic transport) compared to uncoated ZnO NPs. These results seem to imply that using P as a coating invokes different metal translocation strategies, which could be used for other particles, e.g., Cu_3_(PO_4_)_2_. Foliar application of phosphate-containing NPs could potentially be an interesting strategy to change the phyllosphere microbiome to improve the resilience of plants, although this requires further investigation. A better understanding of the interactions between the NP surface and the leaf interface is needed to enhance metal foliar uptake and transport. Enhancing NP uptake for more efficient Zn fruit fortification is essential to tackle crop Zn deficiency and, consequently, improve human health.

## Supporting Information Available

Detailed synthesis and characterization methods for ZnO NPs and ZnO_Ph NPs; details of ICP-MS instrument and analysis, microwave digestion program for NPs, simulated phloem sap composition, Hoagland composition, washing-off solution for exposed leaf composition, details of microwave digestion of pepper plants, Zn content in the pepper seeds, Zn content in the Hoagland solution, Zn content in the sand for plant growth, detailed MeOH digestion of pepper plant tissues, details of spICP-TOFMS instrument and analysis, details of μ-XRF and μ-XANES sample preparation and analysis, reference compounds for Zn μ-XANES analysis, detailed XRD results, TEM/EDS results, and FT-IR results of ZnO NPs and ZnO_Ph NPs, dissolution results of ZnO NPs and ZnO_Ph NPs in MQ water and simulated phloem sap, dry biomass of pepper plants, ^68^Zn uptake by pepper plants and washed-off exposed leaves in percentage, ^68^Zn recovery in percentage, spICP-TOFMS particle counts in all plant tissues, elemental μ-XRF on the DIW control pepper plants, number of POIs performed for Zn μ-XANES in each cell tissue, and LCF for Zn μ-XANES (PDF)
