# Supplementary material for: Effect of a Zinc Phosphate Shell on the Uptake and Translocation of Foliarly Applied ZnO Nanoparticles in Pepper Plants (Capsicum annuum)
Source: Environ Sci Technol. 2024 Feb 10;58(7):3213–23. doi: 10.1021/acs.est.3c08723 (PMC10882962; doi:10.1021/acs.est.3c08723)
Supplement: Supplementary file 1 — es3c08723_si_001.pdf [file es3c08723_si_001.pdf]

Supporting Information

Effect of a Zinc Phosphate Shell on the Uptake and Translocation of Foliarly Applied ZnO Nanoparticles in Pepper Plants (*Capsicum annuum*)

Sandra Rodrigues [a *], Astrid Avellan [b,c], Garret D. Bland [d], Matheus C.R. Miranda [b], Camille Larue [e], Mickaël Wagner [c,e], Diana A. Moreno-Bayona [e], Hiram Castillo-Michel [f], Gregory V. Lowry [d], Sónia M. Rodrigues [a].

[a] Centre for Environmental and Marine Studies (CESAM), Department of Environment and Planning, Universidade de Aveiro, 3810-193, Aveiro, Portugal

[b] Centre for Environmental and Marine Studies (CESAM), Department of Chemistry, Universidade de Aveiro, 3810-193, Aveiro, Portugal

[c] Géosciences-Environnement-Toulouse (GET), UMR 5563 CNRS, UT3, IRD, CNES, OMP, Toulouse, France

[d] Department of Civil and Environmental Engineering, Carnegie Mellon University, Pittsburgh, Pennsylvania 15213, United States

[e] Centre de Recherche sur la Biodiversité et l'Environnement (CRBE), Université de Toulouse, CNRS, IRD, Toulouse INP, Université Toulouse 3 – Paul Sabatier (UT3),Toulouse, France

[f] ESRF, The European Synchrotron, 71 Avenue des Martyrs, CS40220, 38043 Grenoble Cedex 9, France

* Corresponding author. E-mail address: sandra.rodrigues@ua.pt

Number of pages: 26

Number of tables: 9

Number of figures: 14

Materials and Methods

1. ^{68}ZnO-based NP synthesis and characterization

Synthesis

The zinc oxide nanoparticles labeled with ^{68}Zn (ZnO NP) used in this work were synthesized as follows: The precursor Zn Acetate (Zn_Act) labeled with ^{68}Zn was obtained by dissolving 140 mg of metallic ^{68}Zn Powder (Isoflex USA) in 14 mL acetic acid (Sigma Aldrich) at 80°C under stirring for 30 hours (450 rpm). A white powder was obtained by precipitation and then dried at 50 °C for 24h [1].

Two sizes of ZnO NP were synthesized by varying the volume of water used. The smaller size was used for the surface differentiation into Zn phosphate. The previously produced Zn_Act was reduced with methanol (173.09 mg: 3.66 mL ratio), the solution was then refluxed for several minutes at 65°C and water was added (2.47 and 2.74 mL for smaller and bigger particles respectively). A methanol solution containing NaOH (2.71 mL: 65 mg ratio) was added dropwise to the previous solution until obtention of a white precipitate, which was retrieved by centrifugation.

ZnO nanoparticles with phosphate layer (ZnO_Ph NP) were obtained by surface transformation of the smaller ZnO NP (2.47mL water). Na_2HPO_4 solution (150 mg/L^{-1}) was prepared, and the pH was adjusted to 8 with NaOH. ZnO NP (100 mgL^{-1}) were dispersed in the phosphate solution with the aid of an ultrasonic bath for 20 min and incubated at 25 °C in a shaker for 72 h. Samples were centrifuged at 4000g for 1h to separate the solid fraction from the supernatant and washed with milli-q water twice. The separated solids were dried in a desiccator and stored at room temperature for further analysis [2,3].

Characterization

Transmission Electron Microscope (TEM) images were obtained with a Hitachi HT22700B coupled to a dispersive energy spectrometer (EDS), operated at an electron accelerating voltage of 200 kV. Samples were dispersed with milli-q water and deposited on the gold grids of carbon film. Samples were dispersed with milli-q water and deposited on the gold grids of carbon film. Nanoparticle sizes were evaluated using the ImageJ software. The average particle size was obtained by measuring the size of 150 particles.

A Zetasizer Nano-ZS90 (Malvern Instruments, UK) was used to determine nanoparticle surface charge at different pH, an average of 10 readings per sample were measured.

Attenuated total reflection-Fourier transform infrared spectroscopy (ATR-FTIR) was performed to analyze the nanoparticle surface. Each of the samples was placed onto the diamond ATR window of an Avatar 360 Thermo Nicolet spectrometer and scanned over the range of 400–4000 cm−1 with a resolution of 4 cm−1 in transmission mode and expressed as an average of 64 readings [2].

X-ray diffraction analysis (XRD) standards were obtained with Cu-Kα radiation using an Empyrean diffractometer (PANanlytical, The Netherlands). Measurements were made using a step scan program with 0.02° per step and a 5 second acquisition time that was 10 to 60°. XRD data were analyzed using Match 3 (PANanlytical BV Almelo, The Netherlands) for the identification of crystalline phases [4].

The total zinc content was determined by Inductively Coupled Plasma Mass Spectrometry (ICP-MS, Thermo-X Series) according to the following quality control procedures for ICP-MS measurements:

Data acquisition was performed on the Agilent 7700x ICP-MS. A series of 0, 10, 30, 50, 100, 500 and 1000 μg/L calibration standards were used for determining Zn and verified

using an independent certified standard. The standard curve was a linear relationship with r2 at 0.999. Precision was estimated by the relative standard deviation (RSD) of five replicate samples, and it was ≤ 10%. The detection limits (DL) (4 µg/L) were calculated as three times the standard deviations of the blanks (n=10). Samples were digested in a microwave (Table S1) (Speedwave 4, Berghef) by mixing 1 mg of sample with 1.5 mL of HNO_3. The volume was made up to 25 mL of Milli-Q water and measurements were performed in triplicate [5].

Table S1 - Digestion program used in the microwave oven for NPs.

	**Temperature (ºC)**	**Pressure (psi)**	**Ramp (ºC /min)**	**Time (min)**	**Power (watts)**
**1**	180	50	5	15	90
**2**	50	50	1	5	0
**3**	50	50	1	1	0
**4**	50	0	1	1	0
**5**	50	0	1	1	0

2. Dissolved ^{68}Zn release from ZnO NP and ZnO_Ph NP in Milli-Q water and simulated phloem sap

Table S2 - Chemical composition of simulated phloem sap at pH 7.0.

**Chemicals**	**Concentration (mM)**
**Sucrose**	90
**Serine**	11.4
**Aspartate**	9.1
**KCl**	15
**CaCl2**	1.5
**MgSO4**	1.5

**NaCl**	5
**HEPES**	10

3. Pepper seed germination and plant growth

Table S3 - Chemical composition of ¼ strength Hoagland

Chemicals	Concentration (mM)
**KNO_3**	1.29
**$Ca(NO_3).4(H_20)$**	1.20
**$MgSO_4.7(H_2O)$**	0.50
**KH_2PO_4**	0.25
**Na(FeIII)-EDTA**	5.00×10^{-3}
**H_3BO_3**	11.56×10^{-3}
**$MnCl_2$**	2.29×10^{-3}
**$Na_2MoO_4.2H_2O$**	0.12×10^{-3}
**$CuSO_4.5H_2O$**	0.05×10^{-3}
**$ZnSO_4.7H_2O$**	0.19×10^{-3}

5. Assessment of Zn adhesion to pepper exposed leaves by wash-off tests

Table S4 - Chemical composition of the washing solution for the exposed leaves [6]

Chemicals	Concentration (M)
**$CaCl_2$**	5×10^{-4}
**$Ca(NO_3)_2$**	5×10^{-4}
**$MgCl_2$**	5×10^{-4}

**Na_2SO_4**	10^{-4}
**KCl**	10^{-4}

6. Analysis of total Zn concentration inside pepper plant tissues by microwave digestion and ICP-MS analysis

The ratio of sample used for digestion and acids was as follows: 25 – 100 mg sample d.w. : 0.5 mL HNO_3 : 0.25 mL H_2O_2 : 0.25 mL HCl. The digestion consisted of adding 70 % v/v HNO_3 and 30 % v/v H_2O_2 to the dried samples for an overnight pre-digestion (~12 h). The pre-digested samples were submitted to a microwave oven digestion. After cooling down, 37 % v/v HCl was added, samples were submitted once more to a microwave oven digestion, finally obtaining a clear solution indicative of a completely digested sample (the digestion program used is in Table S4).

Table S5 - Digestion program used in the microwave oven for plant tissues.

	Temperature (ºC)	Pressure (psi)	Ramp (ºC /min)	Time (min)	Power (watts)
**1**	175	50	5	10	90
**2**	195	50	5	15	90
**3**	50	0	5	10	90
**4**	50	0	1	10	0
**5**	50	0	1	1	0

Table S6 – Zn concentration in the seeds, Hoagland solution and sand used for plant growth.

	Total Zn
**Pepper seeds**	0.04 ± 0.00 µg Zn / seed
**Hoagland solution**	0.03 ± 0.00 µg Zn / L

**Sand**	2.91 ± 0.66 µg Zn / g

7. Recovery of Zn NPs in pepper plant tissues by Methanol (MeOH) digestion

Zn NPs detection in pepper plant tissues by using Single Particle Inductively Coupled Plasma-Time of Flight Mass-Spectrometry (spICP-TOFMS)

The methanol-based digestion protocol used as by Laughton et al.[7] was performed as follows:

Freshly harvested plant tissues (exposed leaves, remaining leaves, stem, roots, and fruits) along with 10 mM CAPSO buffer at pH 9 (3-(Cyclohexylamino)-2-hydroxy-1-propanesulfonic acid, CAPSO Free Acid, Sigma-Aldrich®) were homogenized with a TissueTearor™ (Biospec Products, Inc.) (1.0 g fresh weight sample: 20 mL CAPSO). The homogenized samples were probe sonicated (550 Sonic Dismembrator™, Fisher Scientific) in ice for 3 min in 10-s intervals. An aliquot was then transferred into a 15 mL centrifuge tube, MeOH (50% v/v) was added to it and shaken at 150 rpm for 1 h. Subsequently, Tween 80® (1.0% v/v) was added, samples were filtered using a DIW pre-rinsed 1.0 µm pore size nylon syringe filter.

Single particle analysis was performed on an inductively coupled plasma time-of-flight mass spectrometer (icpTOF R, TOFWERK AG, Thun, Switzerland). The plasma conditions were optimized during each analysis day, and the instrument was calibrated using a multi-element solution mix with dissolved metal standards in 1% trace-metal grade HNO_3 (Inorganic ventures) within the range of 10 ppt to 5 ppb (iCAP Q/QR Tune Solution, Thermo Scientific). A 50-nm Au NP reference particle (Sigma-Aldrich) and a Au standard solution were used to calculate the transport efficiency [8]. A full description of the instrument and its analytical performance for single particle analysis is previously published [9], and the instrument operational parameters were the same as used in [7].

8. Zn distribution and speciation on pepper fresh tissues using Micro X-ray Fluorescence (µ-XRF) and Micro X-ray Absorption Near-Edge Structure (µ-XANES)

Exposed leaves and stems were embedded in OCT (optimal cutting temperature) resin and flash frozen in liquid nitrogen. Samples were cross-sectioned (20 µm thick) using a Leica cryo-microtome (LN22), placed between two layers of Ultralene film and mounted on an in-house Cu sample-holder immediately after sectioning.

For XANES, an amount of 3 µL of each reference solution (1 µL for NPs) were pipetted between two layers of Ultralene film and mounted on the Cu sample-holder for analysis under cryogenic conditions.

µ-XRF maps were performed using a 9.8 keV incident beam in focused beam mode (beam of 0.3 x 0.7 µm2 with a Kirkpatrick-Baez mirror system) using an in-house DCM monochromator with an average flux of 1.2×10^{10} photon/s. X-ray fluorescence was detected using an SXM fluorescence detector. All energy scans were performed from 9.65 keV to 9.8 keV and maps were recorded with various step sizes (from 0.2 x 0.2 μm^2 to 2 x 2 μm^2).

Table S7 – Reference compounds used for Zn µ-XANES

Reference compound name	Functional group	References for synthesis method
**ZnO NPs**	Zn-O	Dybowska et al. [1]
**ZnO_Ph NPs**	Zn-o-Zn-O-P	Rathnayake et al. [3] and Muthukumaran and Gopalakrishnan, [2]

**Zn-Phytate**	Zn-O-P-R	Asensio et al. [10]
**Zn-Cysteine**	Zn-S-R	Doan et al. [11]
**Zn-Histidine**	Zn-O-R	Provided by Dr. Geraldine Sarret (ISTerre, CNRS & University of Grenoble Alpes, France)
**Zn-Citrate**	Zn-O-R	Purchased from Sigma Aldrich ® (CAS 5990-32-9)

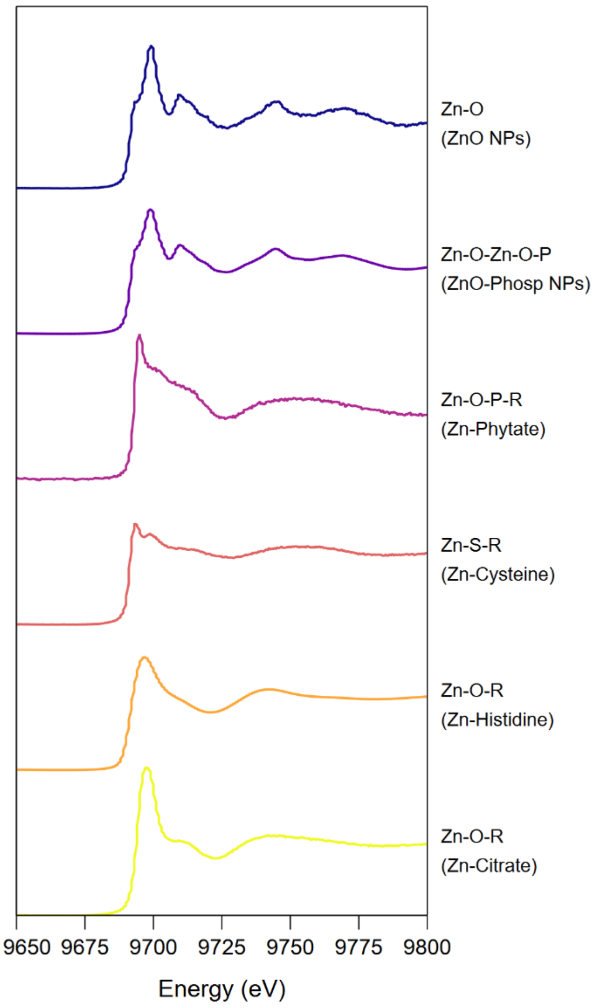

Figure S1 - Reference compounds used for Zn µ-XANES fitting and the simplified bonding environment used for the linear combination fittings. All reference compounds

were analyzed at the ESRF ID21, except for the Zn-Histidine which was provided by Dr. Géraldine Sarret and Zn-Citrate that was purchased from Sigma-Aldrich®.

Results

Nanoparticle surface functionalization and abiotic reactivity

XRD Analysis

XRD analysis showed sharp and intense diffraction peaks at 2θ = 31.8, 34.4, 36.3, 47.6 and 56.7º in the ZnO NP and ZnO_Ph NP samples, which are characteristic of the crystalline hexagonal ZnO NP (pdf 01-071-6424) [3]. It is important to report that it was not possible to observe in ZnO_Ph NP evidence of crystalline anhydrous $Zn_3(PO_4)_2$ in any of the XRD data (Fig. S5). As the XRD technique is sensitive only to crystalline phases, this indicates that the $Zn_3(PO_4)_2$ formed on the surface of nanoparticles at pH 8 is amorphous [3].

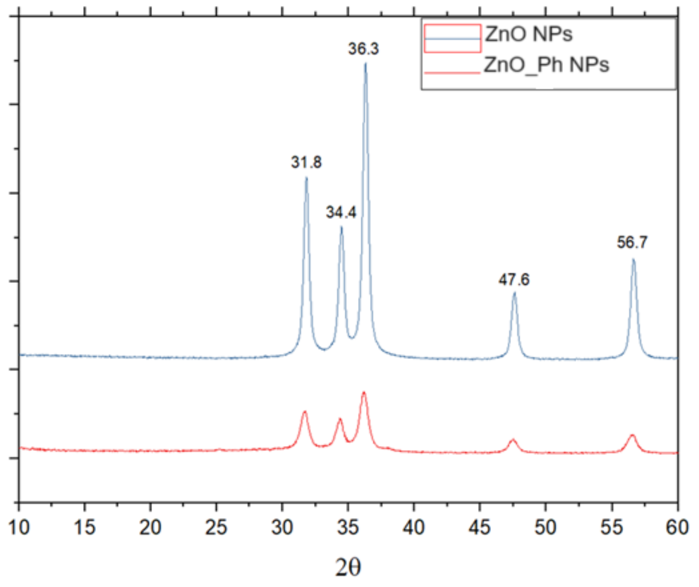

Figure S2 – ZnO NPs and ZnO_Ph NPs XRD diffractogram.

TEM/EDS Analysis

Through the TEM analysis of the ZnO NP nanoparticles (Fig. S1A) it was possible to confirm that the synthesis process was able to form nanoparticles and that they had an average nominal size of 26.36 ± 8.56 nm. Fig. S3B shows a magnification at 600K which makes it possible to see the smooth surfaces of ZnO NP. Through the EDS spectrogram and its ZnO mapping micrograph (Fig. S3C and S3D) it was possible to confirm the presence of O and Zn. It is important to note that the peaks in the characteristic EDS spectrogram for Au and C come from the gold grids with carbon films.

The TEM/EDS analysis of the ZnO_Ph NP nanoparticles (Fig. S4A) showed nanoparticles with an average nominal size of 48.37 ± 11.76 nm. Fig. S4B shows a magnification at 600 K which makes it possible to see rougher surfaces compared to ZnO NP. Through the EDS spectrogram and its ZnO_Ph NP mapping micrograph, it was possible to confirm the presence of O, Zn peaks and a small P peak, which was confirmed by the FT-IR technique (Fig. S6).

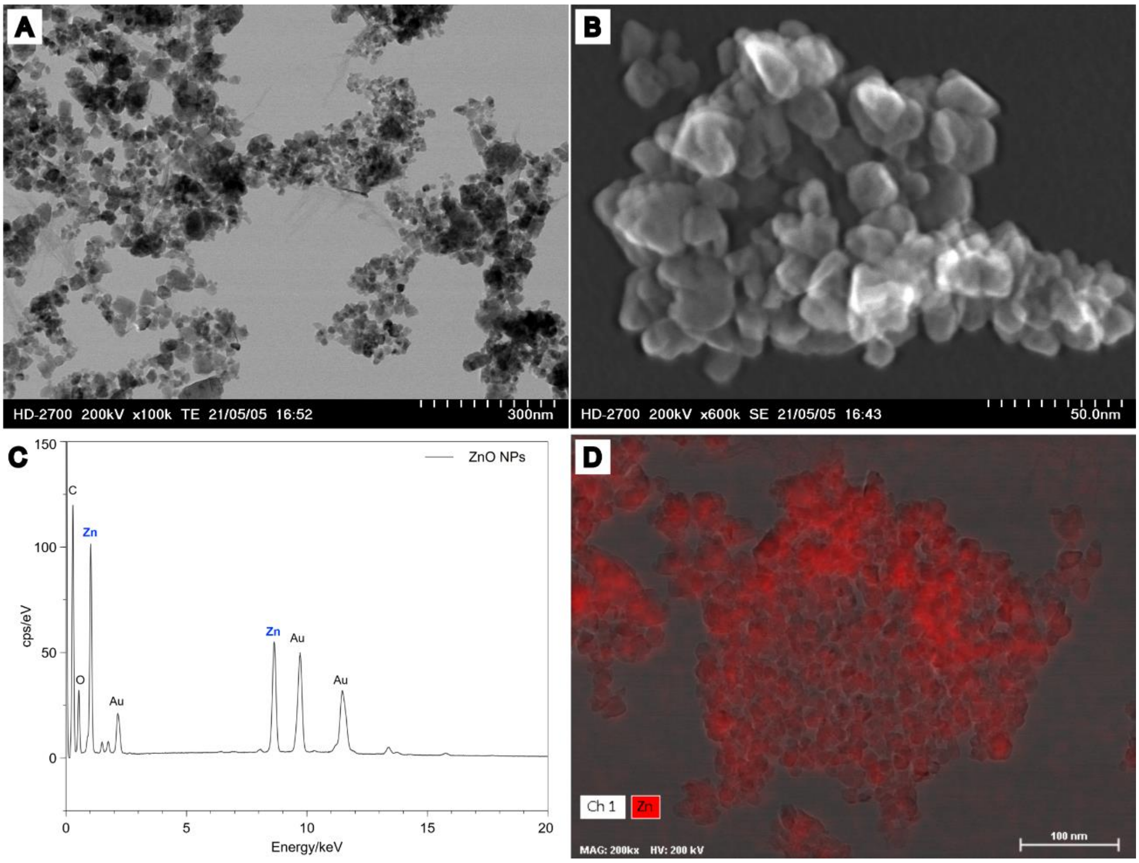

Figure S3 – TEM/EDS analysis of ZnO NP: A) Micrograph at 100Kx magnification; B) Micrograph at a magnification of 600 Kx; C) EDS spectrogram; D) EDS spectrogram Zn mapping micrograph (Zn-red).

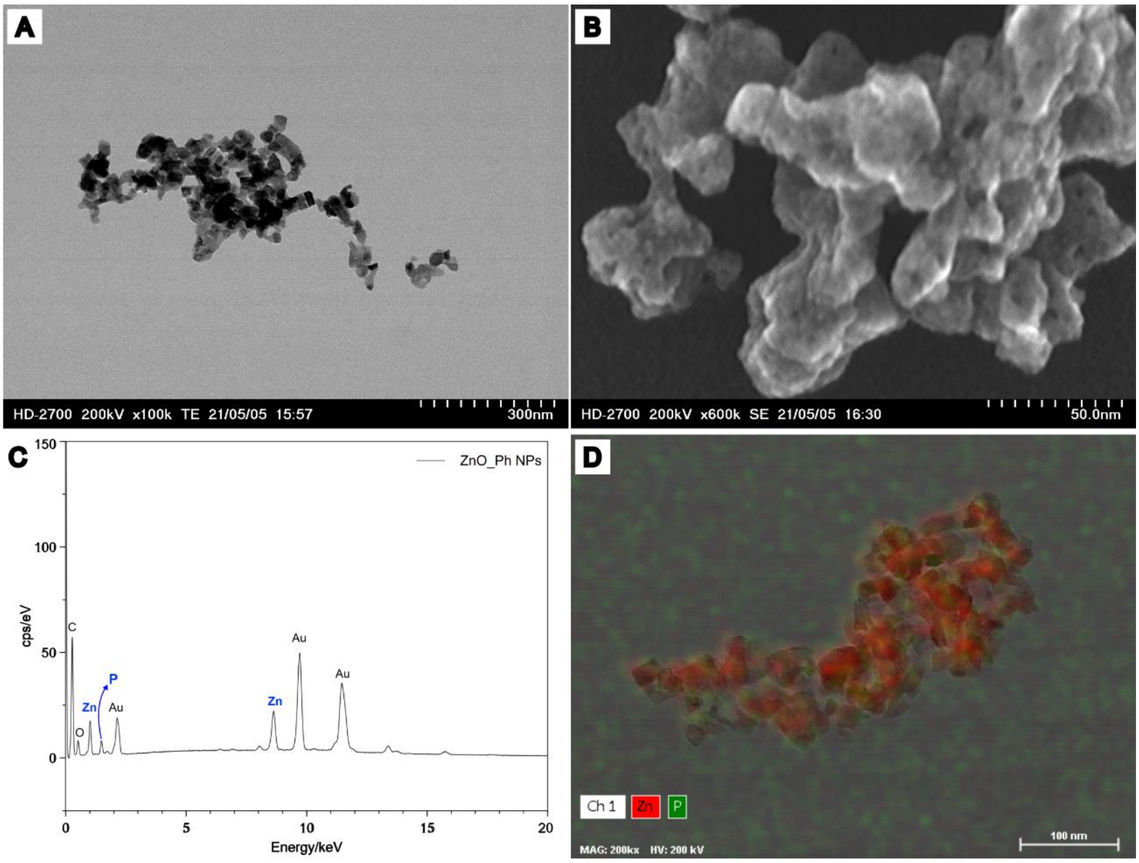

Figure S4 – TEM/EDS analysis of ZnO_Ph NP: A) Micrograph at 100 Kx magnification; B) Micrograph at a magnification of 600 Kx; C) EDS spectrogram; D) and E) EDS spectrogram Zn mapping micrograph (Zn-red, P-green).

FT-IR analysis

FTIR analysis was used to confirm the efficiency of Zn acetate synthesis, as this technique can analyze the characteristic bands of the Zn and acetate groups.

In the ^{68}Zn labeled zinc acetate spectrum (Fig. S5), the following bands were identified: The characteristic bands of asymmetric and symmetrical stretching of the C=O bond in the acetate group were found at 1530 and 1440 cm^{-1}, and two other bands were found at 1030 and 949 cm^{-1} attributed to asymmetric and symmetric stretching of the C-O bond in the acetate group and characteristic bands of Zn-O vibrations at 685 and 610 cm^{-1} [12–14].

It is possible to observe in the FT-IR spectrum of ZnO NP (Fig. S6) the presence of two intense peaks at 1575 and 1418 cm^{-1} with a shoulder at 1441 cm^{-1} observed in the spectrum. A weaker peak at 1336 cm $^{-1}$ is also present. The frequencies of these absorptions are close to those observed for the asymmetric and symmetrical COO stretching movements at 1530 and 1440 cm^{-1} for Zinc Acetate, which is the precursor of ZnO NP synthesis. This similarity between the two ATR-FTIR spectra shown suggests the presence of acetate groups adsorbed on the surface of the ZnO NP [1,15,16]. Other vibrational bands were found at 470 cm^{-1} and 885 cm^{-1} and are characteristic for Zn-O stretching and bending vibration, respectively.

It was possible to observe confirming the $Zn_3(PO_4)_2$ formation on the surface of ZnO NP through the FT-IR technique by observing the characteristic bands of the PO_4^{3-} symmetrical stretching at 1120 cm^{-1} and asymmetrical stretching at 1020 cm^{-1} (Fig. S6). Another characteristic vibrational band of P-O flexion was found at 932 cm^{-1} and the band referring to the Zn-O bond is at 511 cm^{-1}. All of these bands suggest that the desired modification of the ZnO NP surface with $Zn_3(PO_4)_2$ was accomplished.[17].

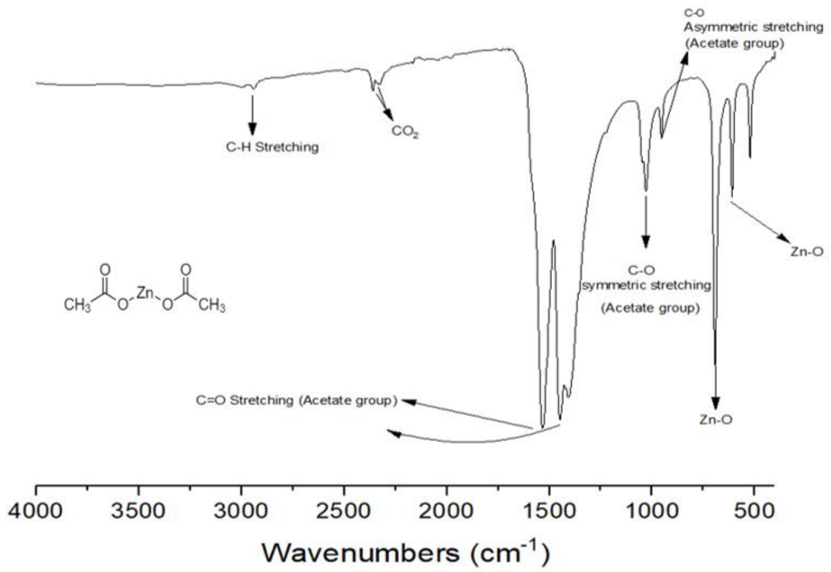

Figure S5 – Zinc acetate spectrum FT-IR.

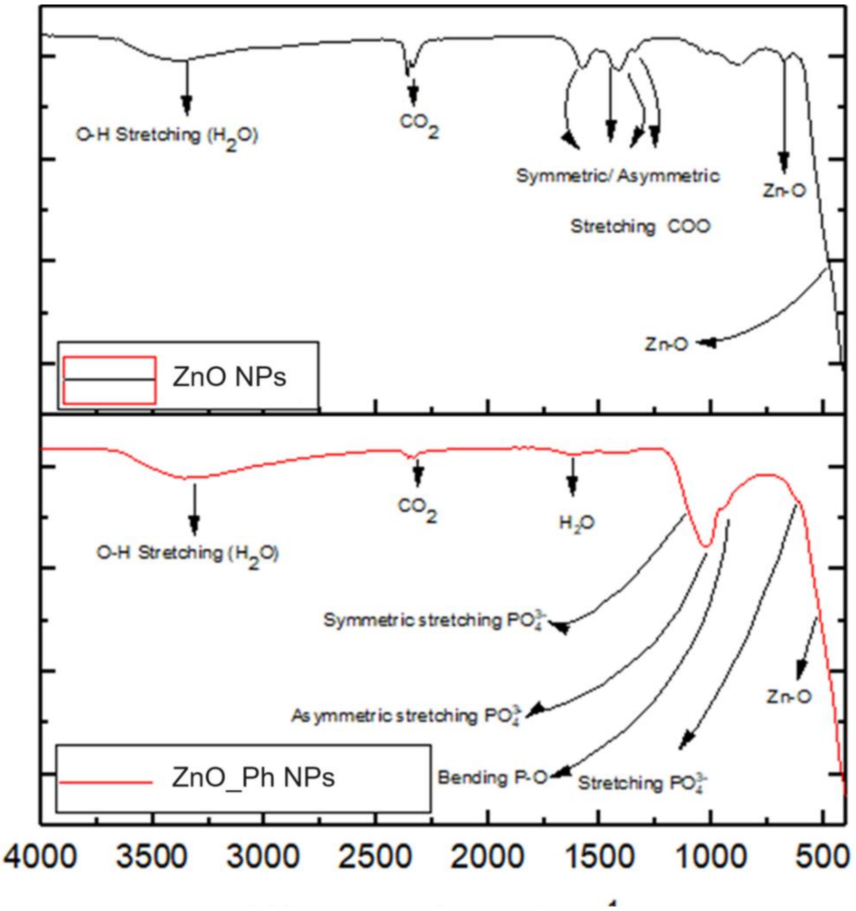

Figure S6 – FTIR spectra of ZnO NP and ZnO_Ph NP.

Dissolution of ZnO NP and ZnO_Ph NPs in MQ water and Simulated phloem sap

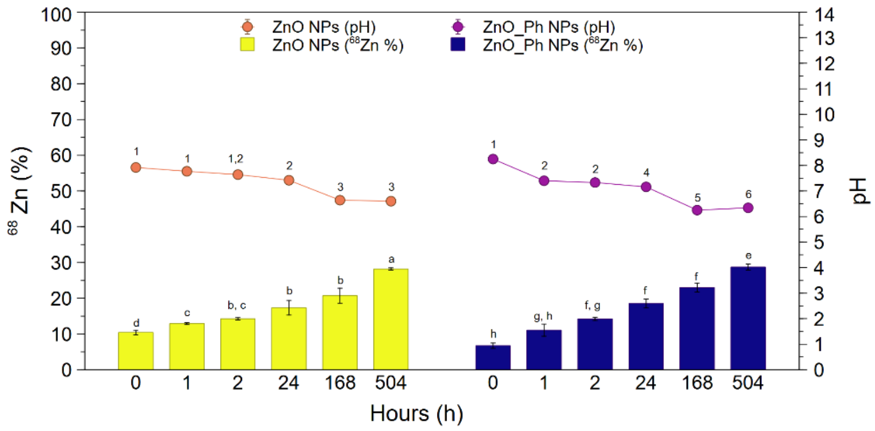

Figure S7 – $^{68}Zn^{2+}$ dissolution as % Zn, released from ZnO NP and ZnO_Ph NP in MQ water, with an initial concentration of 3mg Zn/L. Error bars indicate the standard

deviation of three replicates. Statistically significant differences are indicated with different letters for % Zn and numbers for pH.

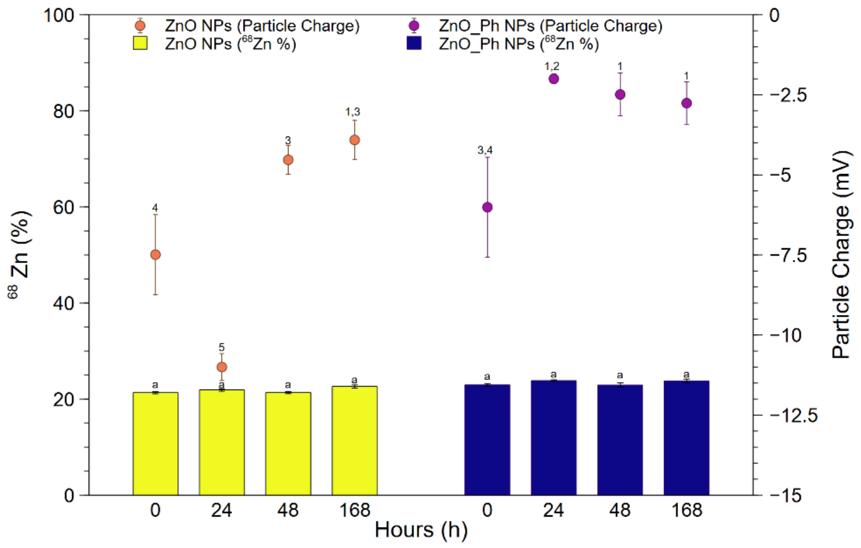

Figure S8 – $^{68}Zn^{2+}$ dissolution as % ^{68}Zn, released from ZnO NP and ZnO_Ph NP and particle charge in simulated phloem sap, with an initial concentration of 3mg Zn/L. Statistically significant differences are indicated with different letters for % Zn and numbers for Particle Charge.

^{68}Zn foliar uptake and *in planta* translocation

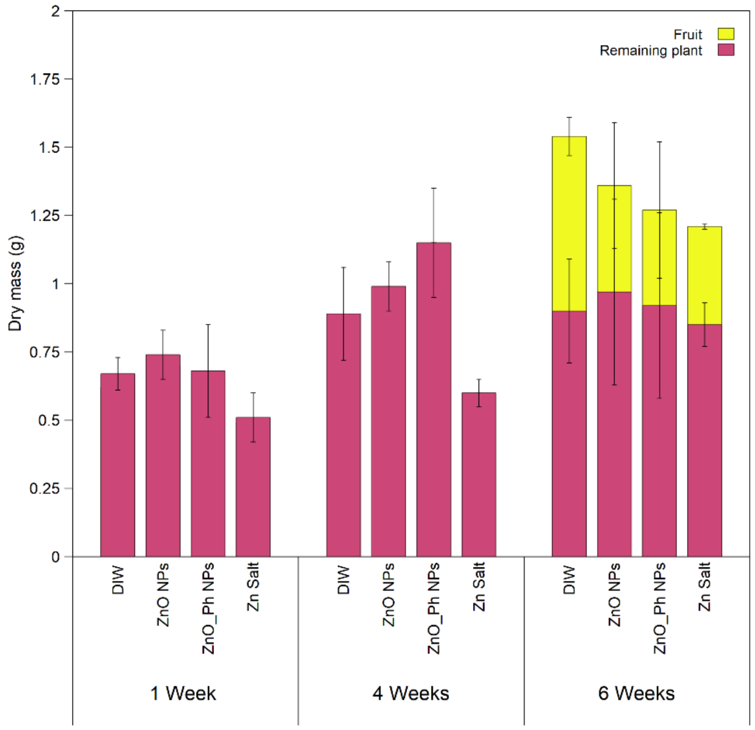

Figure S9 – Dry mass (g) of plants exposed to DIW, ZnO NPs, ZnO_Ph NPs and Zn Salt. Error bars represent the weighted standard deviation of samples from four replicate plants.

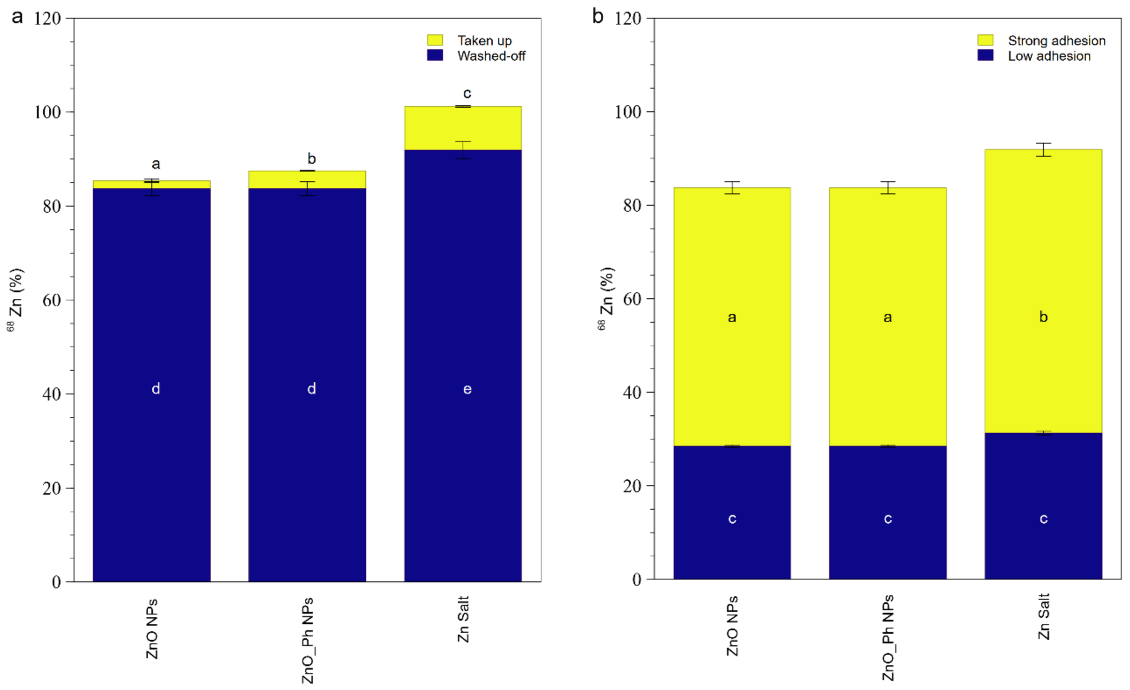

Figure S10 – % ^{68}Zn taken up inside the pepper plants and washed-off both the exposed leaves 1 week after foliar exposure (a), % ^{68}Zn washed-off both exposed leaves as Low adhesion and Strong adhesion to the leaf (b). Error bars represent the weighted standard deviation of samples from four replicate plants. Statistically significant differences of the means are indicated with different letters.

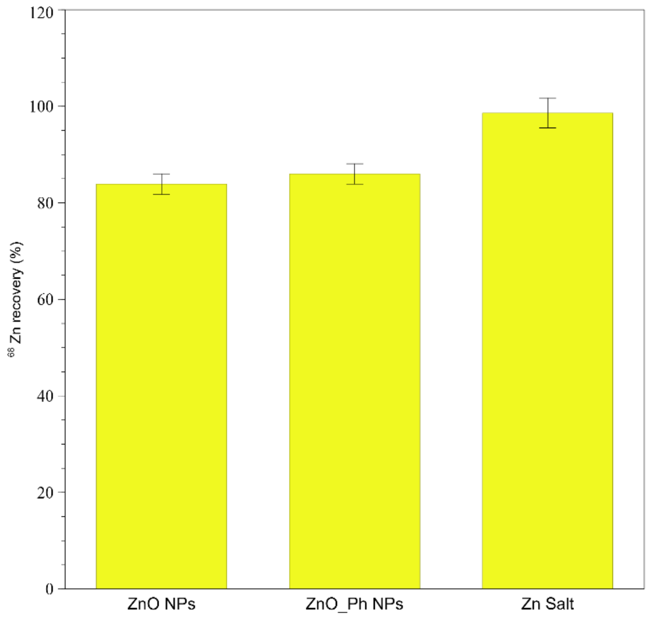

Figure S11 – % ^{68}Zn recovery 1 week after exposure of total ^{68}Zn applied for the plants exposed to ZnO NP, ZnO_Ph NP and Zn Salt. Error bars represent the weighted standard deviation of samples from four replicate plants.

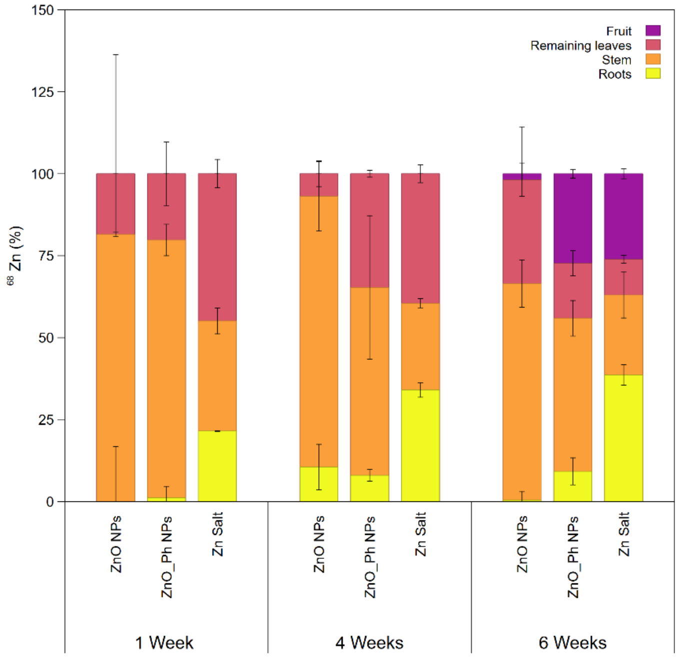

Figure S12 – ^{68}Zn in percentage relative to the ^{68}Zn mass taken up.

^{68}ZnO NPs persistence over time

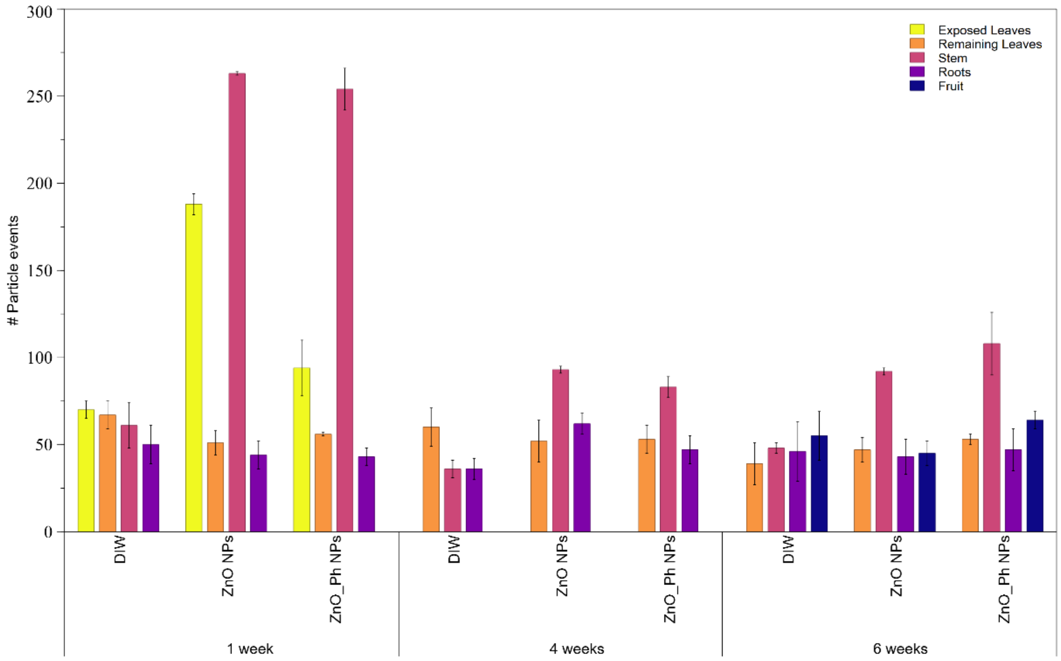

Figure S13 – Particle counts from ICP-TOF-MS measurements in all pepper organs at all timepoints.

Zn *in planta* mobility

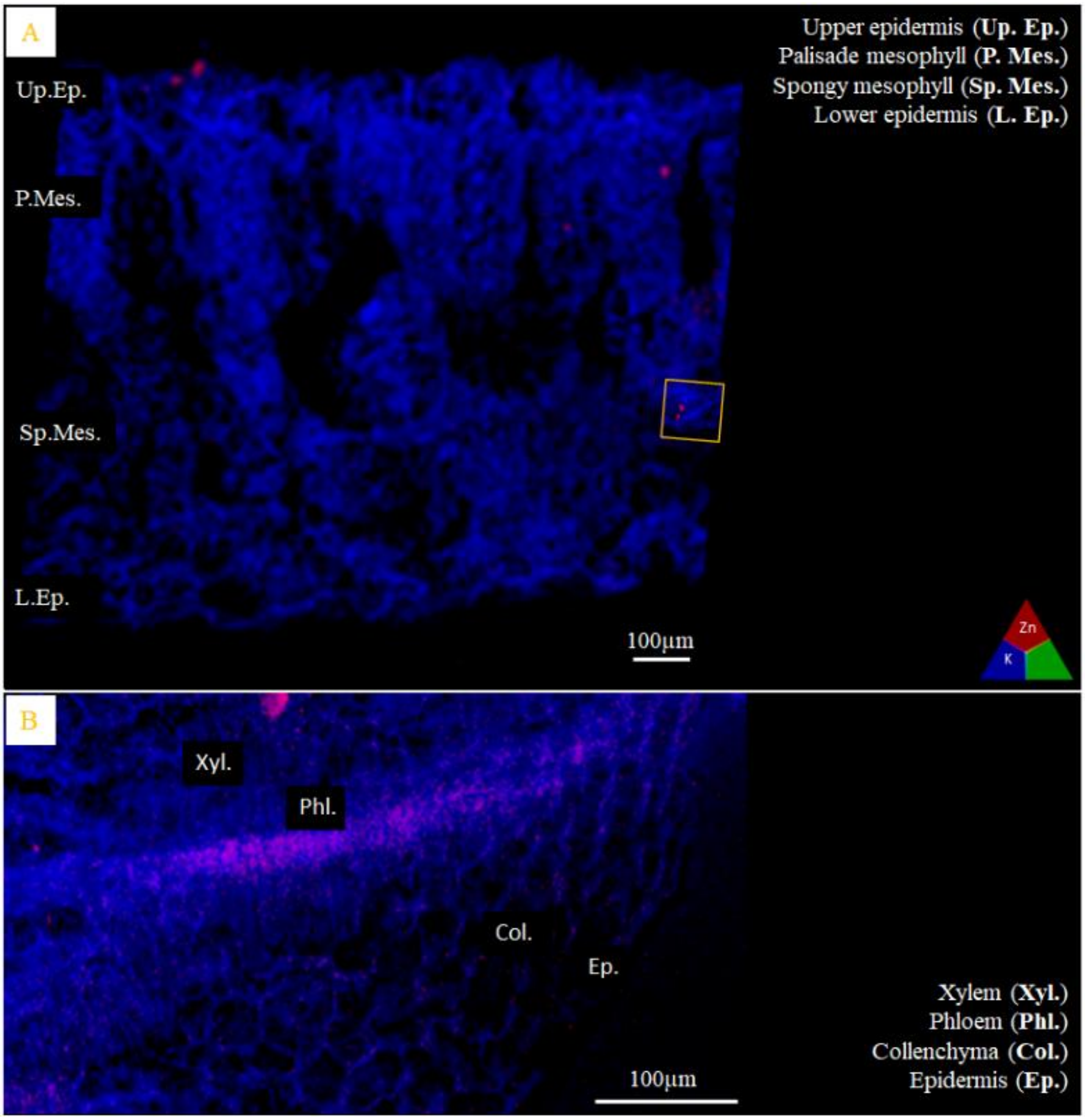

Figure S14 – Elemental µ-XRF on the **DIW control** Pepper plant **7th leaf**: A - 1 week after exposure and **stem near 7th leaf node**: B - 1 week after exposure. Zn is represented in red and K in blue. 2µm x 2µm resolution.

Table S8 – Number of points of interest (POIs) performed in each cell tissue.

Treatment	Time after exposure	Plant tissue	Cell tissue	Nº of POIs
**ZnO NPs**	2 hours	Exposed leaf	Upper epidermis	40
			Palisade mesophyll	34
			Spongy mesophyll	20
	1 week		Upper epidermis	33
			Palisade mesophyll	14
			Spongy mesophyll	34
			Lower epidermis	45
**ZnO_Ph NPs**	2 hours	Exposed leaf	Upper epidermis	14
			Palisade mesophyll	31
			Spongy mesophyll	20
**ZnO NPs**	1 week	Stem	Vasculature	46
**ZnO_Ph NPs**	1 week	Stem	Vasculature	33

Table S9 – Linear Combination Fitting of the µ-XANES spectra done in the different points of interest (POIs) on each cell tissue from the exposed leaves 2 hours, 1 week after exposure and stem 1 week after exposure.

Treatment	Organ	Timepoint	Cell Type	ZnO NPs	ZnO_Ph NPs	Zn-Citrate	Zn-Cysteine	Zn-Histidine	Zn-Phytate	Sum	χ2	Reduced χ2	Rf
**ZnO NPs**	Exposed leaf	2 hours	Upper epidermis	27%	-	-	-	29%	43%	99%	**0.37**	1.33E-03	**1.51E-03**
**ZnO NPs**	Exposed leaf	2 hours	Palisade mesophyll	45%	-	-	-	22%	36%	103%	**0.42**	1.50E-03	**1.50E-03**
**ZnO NPs**	Exposed leaf	2 hours	Spongy mesophyll	-	-	43%	-	19%	42%	104%	**0.22**	8.08E-04	**8.78E-04**
**ZnO NPs**	Exposed leaf	1 week	Upper epidermis	-	-	38%	-	-	71%	108%	**1.25**	4.50E-03	**4.23E-03**
**ZnO NPs**	Exposed leaf	1 week	Palisade mesophyll	-	-	24%	51%	-	28%	103%	**0.37**	1.34E-03	**1.53E-03**
**ZnO NPs**	Exposed leaf	1 week	Spongy mesophyll	-	-	33%	28%	-	41%	103%	**0.19**	6.76E-04	**7.47E-04**
**ZnO NPs**	Exposed leaf	1 week	Lower epidermis	-	-	55%	-	-	46%	100%	**0.29**	1.03E-03	**1.17E-03**
**ZnO_Ph NPs**	Exposed leaf	2 hours	Upper epidermis	-	-	39%	60%	-	-	99%	**1.02**	3.67E-03	**4.74E-03**
**ZnO_Ph NPs**	Exposed leaf	2 hours	Upper epidermis_Nano	-	87%	12%	-	-	-	99%	**1.73**	6.22E-03	**6.83E-03**
**ZnO_Ph NPs**	Exposed leaf	2 hours	Upper epidermis_Non-nano	-	-	48%	51%	-	-	99%	**0.92**	3.30E-03	**4.23E-03**
**ZnO_Ph NPs**	Exposed leaf	2 hours	Palisade mesophyll	-	50%	22%	39%	-	-	112%	**0.96**	3.47E-03	**3.23E-03**
**ZnO_Ph NPs**	Exposed leaf	2 hours	Spongy mesophyll	-	29%	25%	-	-	44%	99%	**0.30**	1.10E-03	**1.24E-03**
**ZnO NPs**	Stem	1 week	Vasculature	-	-	37%	-	-	69%	106%	**0.49**	1.77E-03	**1.73E-03**
**ZnO_Ph NPs**	Stem	1 week	Vasculature	-	-	31%	-	24%	45%	100%	**0.10**	3.65E-04	**4.30E-04**

References

(1) Dybowska, A.; Croteau, M.; Misra, S.; Berhanu, D.; Luoma, S.; Christian, P.; O'Brien, P.; Valsami-Jones, E. Synthesis of Isotopically Modified ZnO Nanoparticles and Their Potential as Nanotoxicity Tracers. *Environ. Pollut.* **2010**, *159*, 266–273. https://doi.org/10.1016/j.envpol.2010.08.032.

(2) Muthukumaran, S.; Gopalakrishnan, R. Structural, FTIR and Photoluminescence Studies of Cu Doped ZnO Nanopowders by Co-Precipitation Method. *Opt. Mater. (Amst).* **2012**, *34* (11), 1946–1953. https://doi.org/https://doi.org/10.1016/j.optmat.2012.06.004.

(3) Rathnayake, S.; Unrine, J. M.; Judy, J.; Miller, A.-F.; Rao, W.; Bertsch, P. M. Multitechnique Investigation of the PH Dependence of Phosphate Induced Transformations of ZnO Nanoparticles. *Environ. Sci. Technol.* **2014**, *48* (9), 4757–4764. https://doi.org/10.1021/es404544w.

(4) Thekkae Padil, V. V.; Černík, M. Green Synthesis of Copper Oxide Nanoparticles Using Gum Karaya as a Biotemplate and Their Antibacterial Application. *Int. J. Nanomedicine* **2013**, *8*, 889–898. https://doi.org/10.2147/IJN.S40599.

(5) Martins, N. C. T.; Avellan, A.; Rodrigues, S.; Salvador, D.; Rodrigues, S. M.; Trindade, T. Composites of Biopolymers and ZnO NPs for Controlled Release of Zinc in Agricultural Soils and Timed Delivery for Maize. *ACS Appl. Nano Mater.* **2020**, *3* (3). https://doi.org/10.1021/acsanm.9b01492.

(6) Kah, M.; Tufenkji, N.; White, J. C. Nano-Enabled Strategies to Enhance Crop Nutrition and Protection. *Nat. Nanotechnol.* **2019**, *14* (6), 532–540. https://doi.org/10.1038/s41565-019-0439-5.

(7) Laughton, S.; Laycock, A.; Bland, G.; von der Kammer, F.; Hofmann, T.; Casman, E. A.; Lowry, G. V. Methanol-Based Extraction Protocol for Insoluble and Moderately Water-Soluble Nanoparticles in Plants to Enable Characterization by Single Particle

ICP-MS. *Anal. Bioanal. Chem.* **2021**, *413* (2), 299–314. https://doi.org/10.1007/s00216-020-03014-8.

(8) Pace, H. E.; Rogers, N. J.; Jarolimek, C.; Coleman, V. A.; Higgins, C. P.; Ranville, J. F. Determining Transport Efficiency for the Purpose of Counting and Sizing Nanoparticles via Single Particle Inductively Coupled Plasma Mass Spectrometry. *Anal. Chem.* **2011**, *83* (24), 9361–9369. https://doi.org/10.1021/ac201952t.

(9) Bland, G. D.; Battifarano, M.; Pradas del Real, A. E.; Sarret, G.; Lowry, G. V. Distinguishing Engineered TiO2 Nanomaterials from Natural Ti Nanomaterials in Soil Using SpICP-TOFMS and Machine Learning. *Environ. Sci. Technol.* **2022**, *56* (5), 2990–3001. https://doi.org/10.1021/acs.est.1c02950.

(10) Asensio, G.; Hernández-Arriaga, A. M.; Martín-del-Campo, M.; Prieto, M. A.; Rojo, L.; Vázquez-Lasa, B. A Study on Sr/Zn Phytate Complexes: Structural Properties and Antimicrobial Synergistic Effects against Streptococcus Mutans. *Sci. Rep.* **2022**, *12* (1), 20177. https://doi.org/10.1038/s41598-022-24300-8.

(11) Doan, M. Y.; Worosz, M. A.; Cheek, G. T. Electrochemical Studies of Zinc/Cysteine Interactions. *ECS Meet. Abstr.* **2017**, *MA2017-01* (37), 1737–1737. https://doi.org/10.1149/ma2017-01/37/1737.

(12) Phoohinkong, W.; Foophow, T.; Pecharapa, W. Synthesis and Characterization of Copper Zinc Oxide Nanoparticles Obtained via Metathesis Process*. *Adv. Nat. Sci. Nanosci. Nanotechnol.* **2017**, *8* (3), 35003. https://doi.org/10.1088/2043-6254/aa7223.

(13) Ullah, N. R.; Thiringer, T.; Karlsson, D. Temporary Primary Frequency Control Support by Variable Speed Wind Turbines— Potential and Applications. *IEEE Trans. Power Syst.* **2008**, *23* (2), 601–612. https://doi.org/10.1109/TPWRS.2008.920076.

(14) Mihaiu, S.; Szilágyi, I. M.; Atkinson, I.; Mocioiu, O. C.; Hunyadi, D.; Pandele-Cusu, J.; Toader, A.; Munteanu, C.; Boyadjiev, S.; Madarász, J.; Pokol, G.; Zaharescu, M.

Thermal Study on the Synthesis of the Doped ZnO to Be Used in TCO Films. *J. Therm. Anal. Calorim.* **2016**, *124* (1), 71–80. https://doi.org/10.1007/s10973-015-5147-2.

(15) Itroutwar, P. D.; Kasivelu, G.; Raguraman, V.; Malaichamy, K.; Sevathapandian, S. K. Effects of Biogenic Zinc Oxide Nanoparticles on Seed Germination and Seedling Vigor of Maize (Zea Mays). *Biocatal. Agric. Biotechnol.* **2020**, *29*, 101778. https://doi.org/https://doi.org/10.1016/j.bcab.2020.101778.

(16) Wu, C.-M.; Baltrusaitis, J.; Gillan, E. G.; Grassian, V. H. Sulfur Dioxide Adsorption on ZnO Nanoparticles and Nanorods. *J. Phys. Chem. C* **2011**, *115* (20), 10164–10172. https://doi.org/10.1021/jp201986j.

(17) Jung, S.-H.; oh, E.; Shim, D.; Park, D.-H.; Cho, S.; Lee, B.; Jeong, Y.; Lee, K.-H.; Jeong, S.-H. Sonochemical Synthesis of Amorphous Zinc Phosphate Nanospheres. *Bull. Korean Chem. Soc* **2009**, *30*. https://doi.org/10.5012/bkcs.2009.30.10.2280.
